# Age-related decline in hippocampal tyrosine phosphatase PTPRO is a mechanistic factor in chemotherapy-related cognitive impairment

**DOI:** 10.1172/jci.insight.166306

**Published:** 2023-07-24

**Authors:** Zhimeng Yao, Hongmei Dong, Jianlin Zhu, Liang Du, Yichen Luo, Qing Liu, Shixin Liu, Yusheng Lin, Lu Wang, Shuhong Wang, Wei Wei, Keke Zhang, Qingjun Huang, Xiaojun Yu, Weijiang Zhao, Haiyun Xu, Xiaofu Qiu, Yunlong Pan, Xingxu Huang, Sai-Ching Jim Yeung, Dianzheng Zhang, Hao Zhang

**Affiliations:** 1 Department of Urology Surgery, and; 2 Department of General Surgery, The First Affiliated Hospital of Jinan University, Jinan University, Guangzhou, Guangdong, China.; 3 Institute of Precision Cancer Medicine and Pathology, School of Medicine, Jinan University, Guangzhou, Guangdong, China.; 4 Department of Pathology, The First People‘s Hospital of Foshan, Foshan, Guangdong, China.; 5 Department of Thoracic Surgery, The First Affiliated Hospital of Jinan University, Jinan University, Guangzhou, Guangdong, China.; 6 Graduate School, Shantou University Medical College, Shantou, Guangdong, China.; 7 Department of Hematology, University Medical Center Groningen, University of Groningen, Groningen, Netherlands.; 8 Department of Pathophysiology, Key Laboratory of State Administration of Traditional Chinese Medicine of the People’s Republic of China, School of Medicine, Jinan University, Guangzhou, Guangdong, China.; 9 Shantou University Mental Health Center,; 10 National Key Disciplines, Department of Forensic and Pathology, and; 11 Center for Neuroscience, Shantou University Medical College, Shantou, Guangdong, China.; 12 Cell Biology Department, Wuxi School of Medicine, Jiangnan University, Wuxi, Jiangsu, China.; 13 The Affiliated Kangning Hospital, Wenzhou Medical University, Wenzhou, Zhejiang, China.; 14 Department of Urology, Guangdong Second Provincial General Hospital, Guangzhou, Guangdong, China.; 15 Minister of Education Key Laboratory of Tumor Molecular Biology, Jinan University, Guangzhou, Guangdong, China.; 16 Gene Editing Center, School of Life Sciences and Technology, ShanghaiTech University, Shanghai, China.; 17 Department of Emergency Medicine and Department of Endocrine Neoplasia and Hormonal Disorders, University of Texas MD Anderson Cancer Center, Houston, Texas, USA.; 18 Department of Biomedical Sciences, Philadelphia College of Osteopathic Medicine, Philadelphia, Pennsylvania, USA.; 19 Institute of Precision Cancer Medicine and Pathology, School of Medicine, and Minister of Education Key Laboratory of Tumor Molecular Biology, Jinan University, Guangzhou, Guangdong, China.

**Keywords:** Aging, Behavior, Neurological disorders, Phosphoprotein phosphatases

## Abstract

Chemotherapy-related cognitive impairment (CRCI) or “chemo brain” is a devastating neurotoxic sequela of cancer-related treatments, especially for the elderly individuals. Here we show that PTPRO, a tyrosine phosphatase, is highly enriched in the hippocampus, and its level is tightly associated with neurocognitive function but declined significantly during aging. To understand the protective role of PTPRO in CRCI, a mouse model was generated by treating *Ptpro^–/–^* female mice with doxorubicin (DOX) because *Ptpro^–/–^* female mice are more vulnerable to DOX, showing cognitive impairments and neurodegeneration. By analyzing PTPRO substrates that are neurocognition-associated tyrosine kinases, we found that SRC and EPHA4 are highly phosphorylated/activated in the hippocampi of *Ptpro^–/–^* female mice, with increased sensitivity to DOX-induced CRCI. On the other hand, restoration of PTPRO in the hippocampal CA3 region significantly ameliorate CRCI in *Ptpro^–/–^* female mice. In addition, we found that the plant alkaloid berberine (BBR) is capable of ameliorating CRCI in aged female mice by upregulating hippocampal PTPRO. Mechanistically, BBR upregulates PTPRO by downregulating miR-25-3p, which directly targeted PTPRO. These findings collectively demonstrate the protective role of hippocampal PTPRO against CRCI.

## Graphical abstract

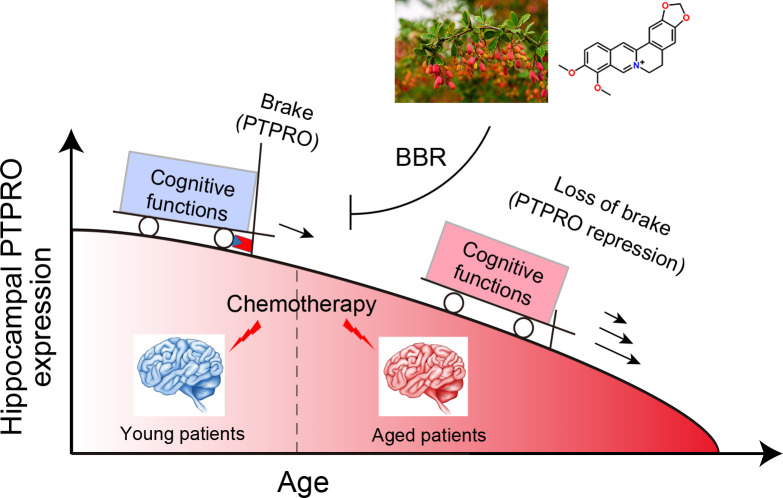

## Introduction

It has been well established that chemotherapy either alone or in combination with other cancer treatment modalities (including targeted reagents and immunotherapies) may induce cognitive dysfunctions that are collectively known as chemotherapy-related cognitive impairment (CRCI), also known as “chemo brain” or “chemo fog” (1). More than 70% of patients who undergo chemotherapy are affected by CRCI (2, 3). The general manifestation of CRCI includes the impairment of memory, concentration, processing speed, executive functioning, attention, learning ability, and language functions (4). Given the rising incidence of cancer with age, CRCI is a particular concern for aged cancer patients undergoing chemotherapy. Indeed, CRCI is more frequent in aged cancer population than reported in studies of younger patients (5). Different hypotheses, including neurotoxic effects, damage to progenitor cells, chronic inflammation, oxidative stress, DNA damage, apoptotic cell death, white matter disruption, mitochondrial disorder, long-term alterations in cerebral blood flow (CBF) and metabolism, and loss of adaptive myelination and alterations of glial cell circuitry have been posed to explain the development of CRCI (6–8). Although chemotherapy is labeled as the causative agent of the CRCI-related cognitive decline, other confounding factors such as genetic and/or epigenetic dysregulation should be considered.

Cancer and neurodegenerative diseases (i.e., Alzheimer disease, AD) are seemingly unrelated diseases, whereas accumulating evidence suggests that the underlying mechanisms as well as some risk factors could be shared by these diseases (9–13). For example, aberrant protein phosphorylation is a critical posttranslational modification for both cancer and neurodegenerative diseases (14–17). The activities of proteins can be affected by their phosphorylation status, which is determined by the ratio of specific kinases and phosphatases. Protein tyrosine phosphate receptor type O (PTPRO) is a member of the R3 subfamily of receptor protein tyrosine phosphatases that dephosphorylates a family of tyrosine kinases (18). Mammalian PTPRO (also known as GLEPP1) was originally cloned from rodent kidney tissue (19). In adult tissues, PTPRO was identified to be highly expressed in the kidney and the brain (20, 21). Nevertheless, its extremely high expression in the brain remains largely enigmatic. High expression of PTPRO orthologs has been found in the brain of mice, zebrafish, and chickens during embryonic and early postnatal stages (20, 22, 23). Of note, the level of PTPRO in mouse brain peaks at embryonic day 16 when neuronal differentiation, axonogenesis, and synaptogenesis are most active (22). In addition, PTPRO is important for functional olfactory bulb, retinal ganglion cells, forebrain, and cerebellum (23–25). It has been proposed that by inhibiting TRKB and RET signaling, PTPRO may also play an essential role in nerve growth factor–induced neurite outgrowth and axonal outgrowth/guidance (26, 27). Evidence from in vitro data suggests that PTPRO may be involved in synapse development, including in neuronal differentiation and axonogenesis, and is important for initiation of synapse formation (28). Results from a genome-wide association study (GWAS) also suggest that PTPRO is highly associated with neurocognitive function (29). However, the role of PTPRO in CRCI and neurocognition is largely unknown.

Berberine (BBR) is an isoquinoline alkaloid originally isolated from *Coptis chinensis* (30). It has been demonstrated that BBR can cross the blood-brain barrier (BBB) and rapidly accumulate in the hippocampal region (31). Although multiple lines of evidence suggest that BBR has neuroprotective effects against cerebral ischemia, depression, schizophrenia, anxiety, and AD (32, 33), the therapeutic effect and relevant mechanism of BBR in CRCI remain to be defined.

In this study, we elucidate the protective role of hippocampal PTPRO in the pathogenesis of CRCI using *Ptpro*^–/–^ female mice treated with a chemotherapeutic reagent, combined with in vivo site-specific restoration of PTPRO by intrahippocampal injection of lentiviral *Ptpro*. We show that hippocampal PTPRO deficiency not only led to CRCI-relevant cognitive impairment in mice, but also conferred therapeutic vulnerability that could be targeted by repurposing BBR. Given that the level of hippocampal PTPRO declines with age and low expression of PTPRO is associated with CRCI development, upregulating PTPRO could be a preventive strategy against CRCI development, especially in elderly patients.

## Results

### PTPRO is highly enriched in the hippocampus and tightly correlated with neuron differentiation, plasticity, and neurogenesis, and hippocampal PTPRO level declines with age.

**Figure 1:**
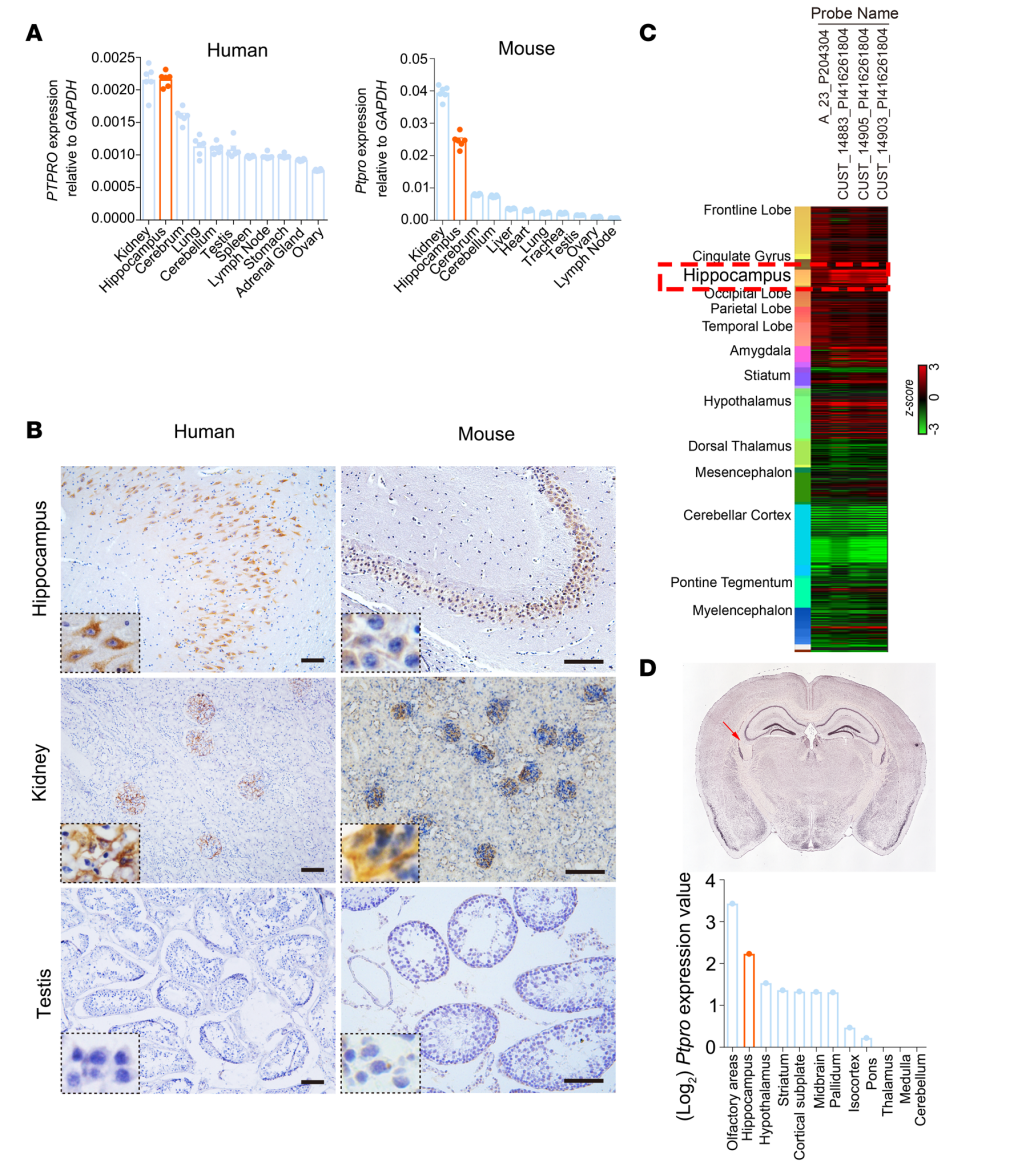
PTPRO is highly expressed in human and mouse hippocampi. (**A**) RT-qPCR analysis of *PTPRO* and *Ptpro* mRNA in different human (*n* = 6 individuals per group with equal sex ratio) and mouse tissues (*n* = 6 mice per group with equal sex ratio), respectively. Results are representative of 3 independent experiments. Error bars: SEM. (**B**) Representative images of IHC staining of PTPRO in different human (left, *n* = 6 individuals per group with equal sex ratio) (Scale bars: 100 μm) and mouse (right, *n* = 6 mice per group with equal sex ratio) (Scale bars: 100 μm) tissues. High expression of PTPRO in the hippocampus (upper panel). The kidney (middle panel) and the testis (bottom panel) were used as controls, which express high and barely detectable levels of PTPRO, respectively. (**C**) The heatmap shows the expression of 4 PTPRO probes in different human brain regions. Gene expression is shown as individually normalized gene expression; red indicates high expression, and green indicates low expression. The red dashed box indicates the hippocampus. Images and data were derived from BrainSpan (http://www.brainspan.org/lcm/search?search_type=user_selections). (**D**) Representative in situ hybridization staining image (upper panel; coronal mouse brain sectional views) and the quantification of the region-specific expression of *Ptpro* in the mouse brain (bottom panel). Red arrows indicate the hippocampus. Images and data were obtained from Allen Mouse Brain Atlas (http://mouse.brain-map.org).

**Figure 2:**
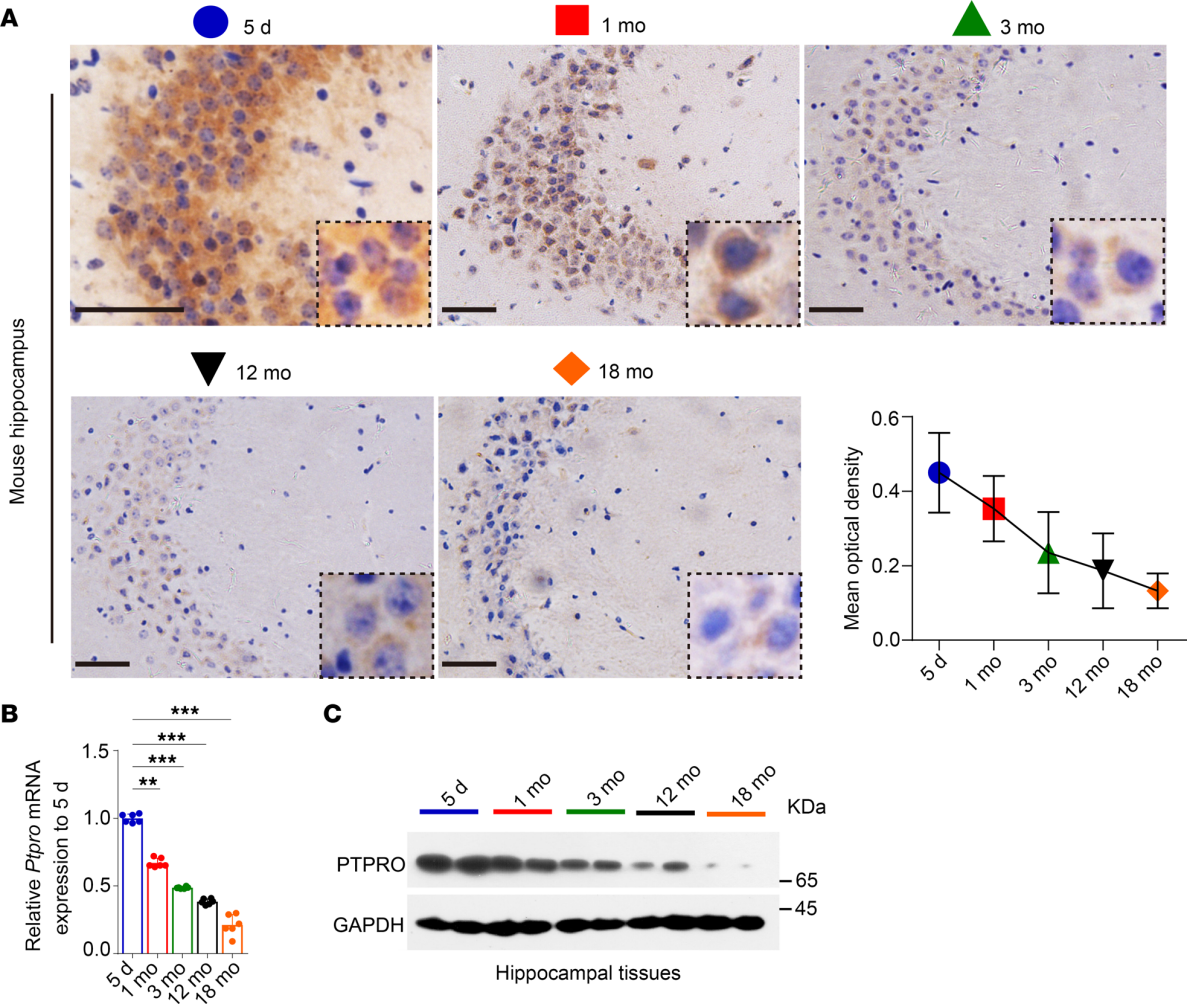
Age-related decrease in PTPRO expression in the hippocampus. (**A**) Representative IHC images of PTPRO in the hippocampal CA3 region of mice at the indicated ages. Boxed areas are enlarged, showing PTPRO expression decreasing with age. Scale bars: 50 μm. Quantification of PTPRO IHC staining in the hippocampal CA3 region of mice at the indicated ages. Error bars: 95% CI. (**B**) The mRNA levels of mice hippocampal *Ptpro* estimated by RT-qPCR. (**C**) The protein levels of mice hippocampal PTPRO estimated by immunoblotting. *n* = 6 mice per age group with equal sex ratio (**A**–**C**). Representative data from 1 of 3 independent experiments are shown in **B** and **C**. Error bars: SEM. **P < 0.01, ***P < 0.001 by 1-way ANOVA followed by a Tukey-Kramer post hoc test (**B**).

The hippocampus plays a central role in memory, learning, and cognitive ability (34). Accumulating evidence suggests that CRCI and AD, both age-related diseases, may share similar genetic variations, molecular pathways, and risk factors (35–38). Until now, there has been no public functional genomics database available to study molecular pathological alterations in CRCI hippocampi; therefore, we conducted gene set enrichment analyses (GSEAs) of the NCBI Gene Expression Omnibus (GEO) GSE29378 data set, which includes hippocampi from 31 AD patients, and found that *PTPRO* expression in the human hippocampus is highly correlated with gene signatures related to neuronal differentiation, synaptic plasticity, neuronal recognition, neurogenesis, neuronal generation, and dendritic formation (Supplemental Figure 1; supplemental material available online with this article; https://doi.org/10.1172/jci.insight.166306DS1). In addition, a different GEO database (GSE14938) indicated that *PTPRO* is highly expressed in the hippocampus in addition to the kidney (Supplemental Figure 2A). Reverse transcription–quantitative PCR (RT-qPCR) assays of different human and mouse tissues demonstrated that *PTPRO* is indeed highly expressed in hippocampi (Figure 1A), whereas immunohistochemistry (IHC) revealed that hippocampal CA3 pyramidal neurons exhibits the strongest PTPRO staining (Figure 1B). These data are also consistent with that in the Allen Brain Atlas (Figure 1, C and D, and Supplemental Figure 2B). Of note, the data extrapolated from the Brain EXPression Database (BrainEXP) and GEO showed that PTPRO levels in postnatal hippocampi negatively correlated with aging (Supplemental Figure 3, A–E), and these findings were also corroborated in mouse hippocampi by IHC, RT-qPCR, and immunoblotting (Figure 2, A–C). In contrast, there was no detectable age-dependent change in the levels of mouse kidney PTPRO (Supplemental Figure 4, A and B). These findings altogether indicate that the expression level of hippocampal PTPRO declines with age, and suggest that PTPRO may play an indispensable role in neurocognitive-related functions.

### Ptpro deficiency increases doxorubicin-induced CRCI in 3-month-old mice.

**Figure 3:**
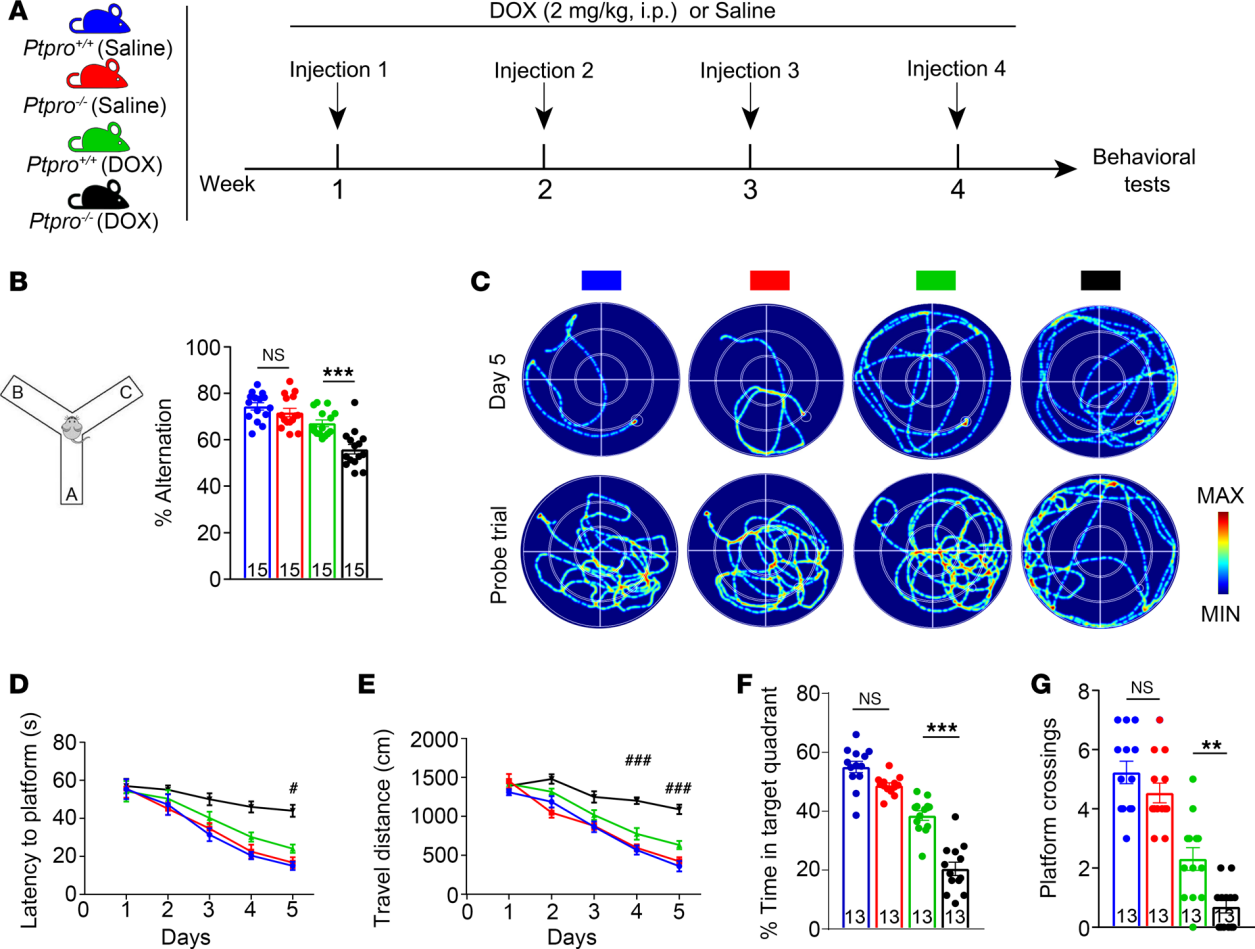
*Ptpro* deletion increases DOX-induced CRCI in 3-month-old female mice. (**A**) The scheme of the treatments. (**B**) The spontaneous alternation of the Y-maze test. (**C**) Representative traces of swimming plot in the MWM test. (**D**) The time to reach the submerged platform. (**E**) The distances traveled before reaching the submerged platform. (**F**) The time spent in the target quadrant. (**G**) The number of crossings before reaching the target location. ^#, ###^Indicate DOX-treated *Ptpro^+/+^* mice vs. DOX-treated *Ptpro^–/–^* mice and **^,^ ***Indicate DOX-treated *Ptpro^+/+^* mice vs. DOX-treated *Ptpro^–/–^* mice; *n* = 13 or 15 per group. Error bars: SEM. NS, not significant; **P < 0.01, ***P < 0.001; ^#^*P* < 0.05, ^###^*P* < 0.001 by 2-way ANOVA (**B**, **F**, and **G**) or 3-way ANOVA (**D** and **E**) followed by a Tukey-Kramer post hoc test. All values and statistical analysis of behavioral experiments are provided in Supplemental Table 1.

Given that hippocampal PTPRO expression is tightly associated with neurocognitive-related functions and decreases with age, we hypothesized that the age-related decrease in hippocampal PTPRO might be a mechanistic factor for CRCI in elderly cancer patients. To understand the protective role of PTPRO in CRCI, we selected administration of doxorubicin (DOX), which is one of the most active agents for treatment of breast cancer, once a week for 4 weeks to induce a CRCI model in 3-month-old *Ptpro*^+/+^ and *Ptpro*^–/–^ female mice (Figure 3A). DOX can cause severe cognitive impairment in patients through a variety of mechanisms. Notably, the hippocampus is the most likely brain region affected in DOX-induced CRCI (39–41).

To determine PTPRO’s neurocognitive role in the CRCI mouse model, we evaluated the performance of *Ptpro*^+/+^ and *Ptpro*^–/–^ female mice in the Y maze and the Morris water maze (MWM). During a 10-minute session of the Y-maze test, the *Ptpro*^+/+^ and *Ptpro*^–/–^ mice in the saline treatment group did not show any observable difference in the proportion of alternation. However, the *Ptpro*^–/–^ mice in the DOX treatment group decreased their alternation rate significantly compared with the age-matched *Ptpro*^+/+^ mice (Figure 3B). In addition, DOX-treated *Ptpro*^–/–^ mice exhibited obvious defects in cognitive abilities, as measured by latency to reach the platform (Figure 3, C and D), distance traveled (Figure 3E), time in quadrants (Figure 3F), and the number of platform crossings (Figure 3G) in the MWM test. It has been reported that blood pressure, cerebral hemodynamics, and the integrity of the BBB are closely related to brain/hippocampal function, and DOX can increase blood pressure, reduce CBF, and destroy the BBB, thus promoting cognitive impairment (42–45). We monitored and evaluated these physiological indicators in DOX-induced CRCI at the end of the trial. As shown in Supplemental Figure 5, the *Ptpro*^+/+^ and *Ptpro*^–/–^ mice in either the saline-treated or DOX-treated group did not show any significant difference in their blood pressure, CBF, and BBB integrity, indicating that PTPRO plays a protective role against cognitive dysfunction in DOX-induced CRCI through mechanisms other than influencing these physiological indicators.

In addition, due to the CRCI research using tumor-bearing animals to mimic humans with newly diagnosed cancer is necessary to screen potential drug candidates against CRCI (46), we investigated the potential relevance of PTPRO to cognitive function in tumor-bearing mice. We transplanted tumors derived from MMTV-PyMT transgenic mice orthotopically into the mammary fat pads of *Ptpro*^+/+^ and *Ptpro*^–/–^ mice followed by DOX treatment to establish tumor-bearing mouse model of CRCI (Supplemental Figure 6A). DOX treatment significantly inhibited tumor growth (Supplemental Figure 6B). Tumor volumes in the saline- or DOX-treated group were comparable, suggesting that *Ptpro* deficiency in host mice does not affect tumor growth (Supplemental Figure 6B). These findings are consistent with previous studies showing that tumor-bearing mice displayed cognitive impairments compared with the normal mice (46) (Supplemental Figure 6, C–I). In addition, saline treatment did not affect cognitive function and hippocampal synaptic plasticity, as measured by MWM testing and long-term potentiation (LTP) (Supplemental Figure 6, C–I). On the other hand, cognitive abilities and hippocampal synaptic plasticity were significantly affected when the *Ptpro*^–/–^ tumor-bearing mice were treated with DOX (Supplemental Figure 6, C–I). These data collectively indicate that PTPRO has comparable neuroprotective roles in both healthy and tumor-bearing mice.

### Ptpro deficiency reduces neuronal survival and neurogenesis and leads to neurodegeneration in DOX-induced CRCI.

**Figure 4:**
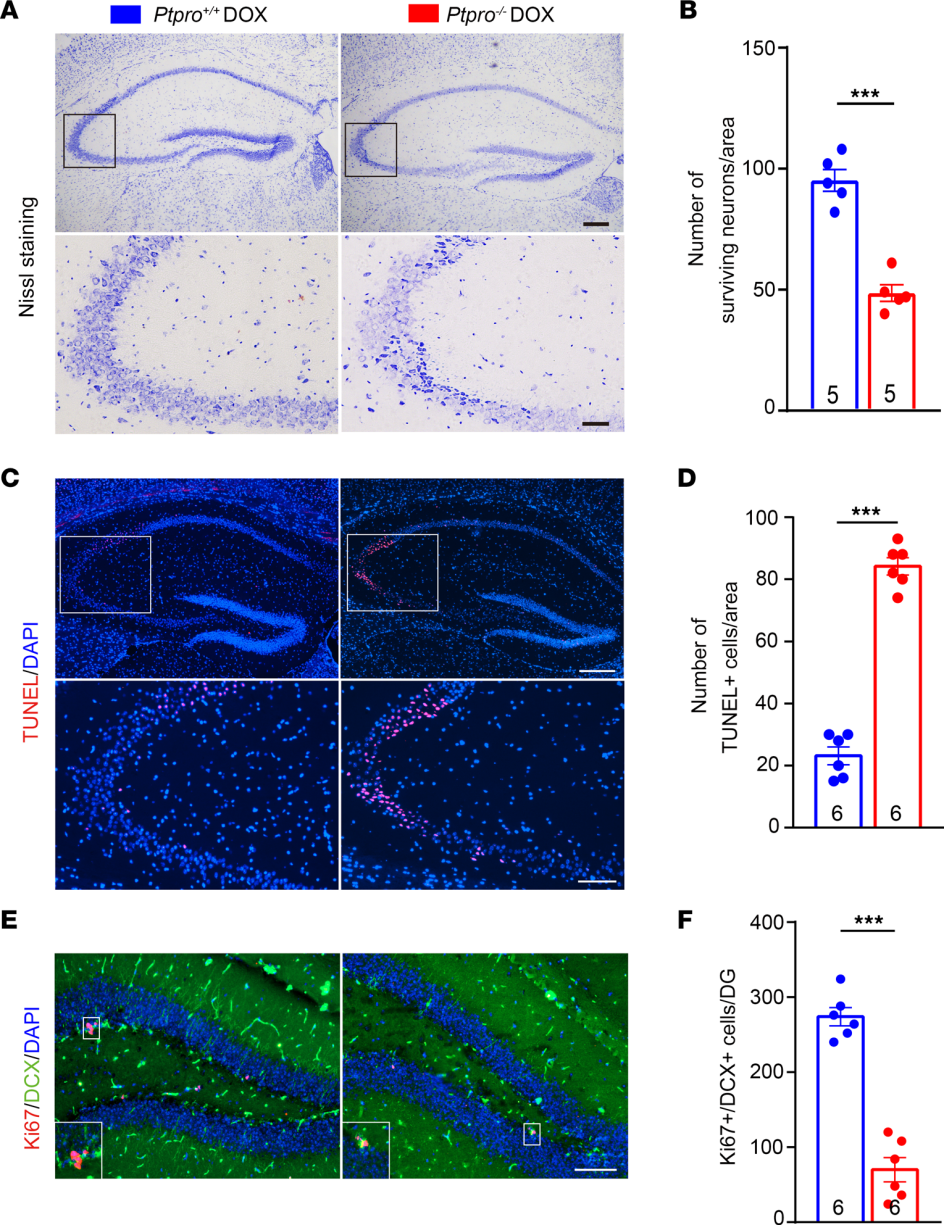
*Ptpro^–/–^* female mice treated with DOX exhibit more severe neurodegeneration and impaired neurogenesis in the hippocampi following DOX treatment. (**A**) Nissl staining of the hippocampi (upper panel) and hippocampal CA3 region (bottom panel) in *Ptpro*^+/+^ and *Ptpro^–/–^* mice. Scale bars: 200 μm (upper panel), 50 μm (bottom panel). (**B**) Quantification of surviving neurons in the hippocampal CA3 region of *Ptpro*^+/+^ and *Ptpro^–/–^* mice. *n* = 5 mice per genotype. (**C**) TUNEL staining of the hippocampi (upper panel) and hippocampal CA3 region (bottom panel) in *Ptpro*^+/+^ and *Ptpro^–/–^* mice. Scale bars: 250 μm (upper panel), 100 μm (bottom panel). (**D**) Quantification of TUNEL-positive hippocampal CA3 cells in *Ptpro*^+/+^ and *Ptpro^–/–^* mice. *n* = 6 mice per genotype. (**E**) Representative images of immature (DCX^+^) and proliferating (Ki67^+^) cells in the hippocampi. Magnified views of areas in the white box are shown in the bottom left corner of each image. Scale bars: 100 μm. (**F**) Quantification of proliferating immature cells in the hippocampi. DG, dentate gyrus. *n* = 6 mice per genotype. These results are representative of 3 independent experiments. Error bars: SEM. ***P < 0.001 by 2-sided Student’s *t* test.

To further assess the effects of PTPRO in DOX-induced CRCI, we conducted Nissl staining to observe the neuronal morphology and quantity of hippocampal CA3 regions in *Ptpro^+/+^* and *Ptpro*^–/–^ female mice treated with DOX. As shown in Figure 4A, the neurons were obviously shrunken and weakly stained in the *Ptpro*^–/–^ mice treated with DOX, indicating diffusely deteriorated neurons and increased neuronal loss. The number of surviving CA3 neurons in the *Ptpro*^–/–^ mice treated with DOX was decreased compared with the wild-type (WT) controls (Figure 4B). TUNEL staining also showed increased apoptosis in the CA3 region of the *Ptpro*^–/–^ mice treated with DOX (Figure 4, C and D). These data suggest that the *Ptpro* deletion leads to an increased susceptibility for hippocampal neuronal death in mice treated with DOX. Additionally, the number of Ki67 (proliferation marker) and doublecortin (DCX; an immature progenitor cell marker) double-labeled neurons (indicating proliferating immature neurons) in the subgranular zone (SGZ) of the dentate gyrus significantly decreased in the *Ptpro*^–/–^ mice treated with DOX (Figure 4, E and F).

### Loss of Ptpro results in the dysregulation of synaptic plasticity in DOX-induced CRCI.

**Figure 5:**
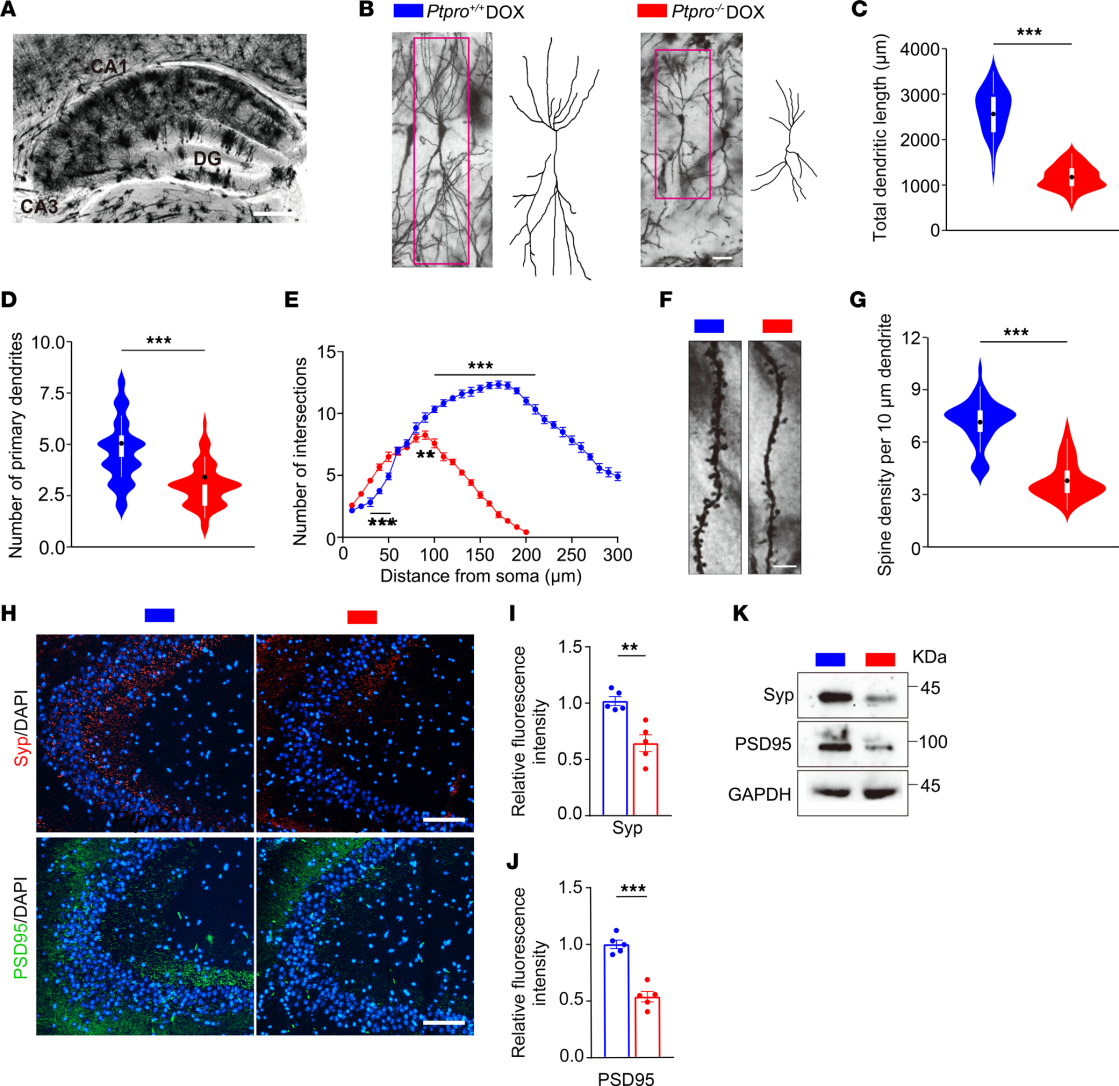
*Ptpro^–/–^* female mice display more severe defects in dendritic spine morphogenesis and synaptic function in the hippocampi following DOX treatment. (**A**) Representative image of Golgi-stained hippocampal sections from *Ptpro*^+/+^ mice treated with DOX. Scale bar: 500 μm. DG, dentate gyrus. (**B**) Representative images and drawings of Golgi-stained hippocampal CA3 pyramidal neurons from *Ptpro*^+/+^ (left panel) and *Ptpro^–/–^* mice (right panel). Scale bar: 50 μm. (**C** and **D**) Quantification of the total dendritic length and primary dendrites of CA3 pyramidal neurons from the *Ptpro*^+/+^ and *Ptpro^–/–^* mice. *n* = 4 per genotype. At least 10 cells were analyzed per mouse. (**E**) Sholl’s analysis of the complexity of CA3 pyramidal neurons the *Ptpro*^+/+^ and *Ptpro^–/–^* mice. *n* = 4 per genotype. At least 10 cells were analyzed per mouse. (**F**) Representative photomicroscopy images of Golgi-stained dendrites of CA3 pyramidal neurons from the *Ptpro*^+/+^ and *Ptpro^–/–^* mice. Scale bars: 5 μm. (**G**) Quantitative analysis of spine densities in CA3 pyramidal neurons from the *Ptpro*^+/+^ and *Ptpro^–/–^* mice. *n* = 4 per genotype; an average of 5 dendrites of CA3 pyramidal neurons were analyzed per mouse. (**H**) Representative immunofluorescence images of synaptophysin (Syp) and PSD95 in hippocampal CA3 sections from *Ptpro*^+/+^ and *Ptpro^–/–^* mice. Scale bars: 100 μm. (**I**) Quantification analysis of the average fluorescence intensity of Syp in hippocampal CA3 sections from *Ptpro*^+/+^ and *Ptpro^–/–^* mice. *n* = 5 per genotype. (**J**) Quantification analysis of the average fluorescence intensity of PSD95 in hippocampal CA3 sections from *Ptpro*^+/+^ and *Ptpro^–/–^* mice. *n* = 5 per genotype. (**K**) Immunoblotting of Syp and PSD95 in the hippocampi of *Ptpro*^+/+^ and *Ptpro^–/–^* mice. These results are representative of 3 independent experiments. Error bars: SEM. **P < 0.01, ***P < 0.001 by 2-sided Student’s *t* test (**C**, **D**, **G**, **I**, and **J**) or 2-way ANOVA followed by Bonferroni’s multiple-comparison test (**E**).

Synaptic plasticity is important to cognitive functions and its dysregulation is associated with many neuropsychiatric disorders, including cognitive dysfunctions such as AD (47–49). Golgi staining was performed, using hippocampal samples from DOX-treated *Ptpro*^+/+^ and *Ptpro*^–/–^ female mice, to examine the morphology of CA3 pyramidal neurons. In the hippocampal region (Figure 5A), the total dendritic length and the numbers of primary dendrites of CA3 pyramidal cells in *Ptpro*^–/–^ mice were lower than in WT controls following DOX treatment (Figure 5, B–D). Moreover, Sholl’s analysis revealed markedly reduced dendritic branching of CA3 neurons in *Ptpro*^–/–^ mice compared with WT controls (Figure 5E). Apical dendrites of *Ptpro*^–/–^ CA3 pyramidal neurons displayed an approximately 50% decreased spine density compared with WT controls when treated with DOX (Figure 5, F and G). Synaptophysin (Syp, an essential presynaptic vesicle membrane protein) and postsynaptic density protein 95 (PSD95, a postsynaptic scaffold protein) are closely related to hippocampal synaptic plasticity and cognitive function. Immunofluorescent staining revealed reduced Syp and PSD95 intensity in CA3 of the hippocampi in *Ptpro*^–/–^ mice compared with WT controls when treated with DOX (Figure 5, H–J). These results were further validated by immunoblotting for Syp and PSD95 (Figure 5K). Thus, the DOX-treated *Ptpro*^–/–^ mice, compared with WT controls, display more dramatically severe cognitive deficits that correlate with alterations in synaptic plasticity of CA3 hippocampal neurons.

### Hippocampal Ptpro deficiency is associated with abnormal activation of SRC/EPHA4 in DOX-induced CRCI.

PTPRO is a single-pass transmembrane protein with an extracellular domain containing 8 fibronectin type III–like domains and an intracellular protein tyrosine phosphatase domain (19). Given its well-defined function as a tyrosine phosphatase, we focused our attention on the 40 tyrosine kinases that have been reported to be associated with neurocognition. Of note, 3 of them — SRC, EPHA4, and EPHB2 — that are not only related to neurotoxicity but also serve as PTPRO substrates (Supplemental Figure 7 and Supplemental Table 3), and these kinases are highly expressed in both human and mouse hippocampi (Supplemental Figure 8). Furthermore, results from GSEA of human and mouse data sets revealed that activated SRC, EPHA4, and EPHB2 were closely associated with AD (Supplemental Figure 9). Consistently, these kinases in mice are positively correlated with neuronal death and negatively correlated with neurogenesis (Supplemental Figure 10).

**Figure 6:**
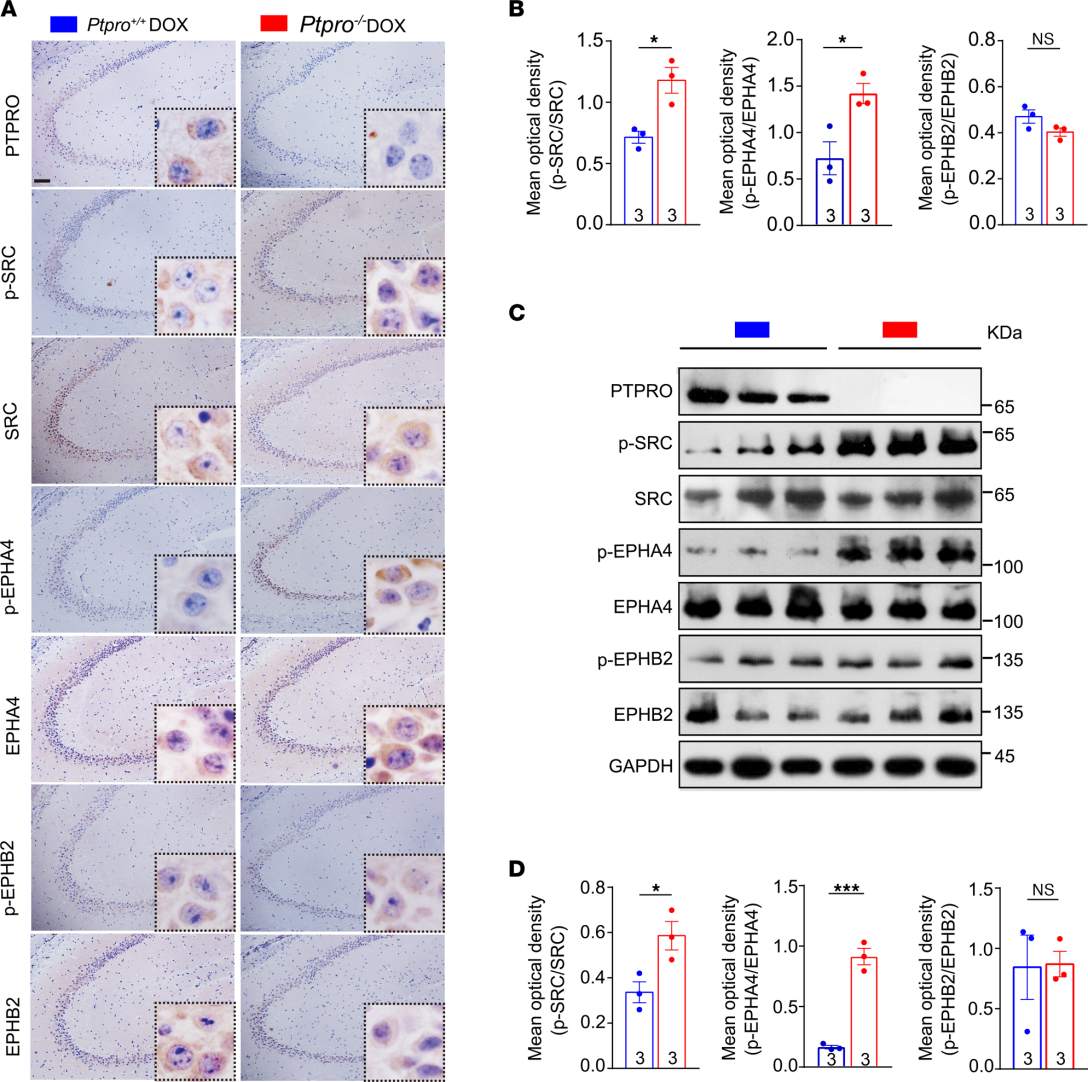
Amelioration of CRCI by hippocampal PTPRO is associated with inactivation of the SRC/EPHA4 axis. (**A**) Representative IHC images of PTPRO, p-SRC, SRC, p-EPHA4, EPHA4, p-EPHB2, and EPHB2 in the hippocampi. Scale bar: 50 μm. (**B**) The mean optical density of p-SRC/SRC, p-EPHA4/EPHA4, and p-EPHB2/EPHB2 in the hippocampi. (**C**) Immunoblotting of PTPRO, p-SRC, SRC, p-EPHA4, EPHA4, p-EPHB2, and EPHB2 in the hippocampi under CRCI. Data are representative of 3 independent experiments. (**D**) The mean optical density of p-SRC/SRC, p-EPHA4/EPHA4, and p-EPHB2/EPHB2 in the hippocampi. Error bars: SEM. NS, not significant; *P < 0.05, ***P < 0.001 by 2-sided Student’s *t* test.

To determine whether these kinases are indeed regulated by PTPRO during DOX-induced CRCI, we quantified the levels of phosphorylated forms of these enzymes when the mice were treated with DOX. The levels of phosphorylated SRC and EPHA4 were significantly elevated in the hippocampi of the *Ptpro^–/–^* female mice when they were treated with DOX (Figure 6). Since phosphorylation of EPHB2 did not appear to be affected by *Ptpro* deletion (Figure 6), we are inclined to conclude that PTPRO-repressed SRC and EPHA4 phosphorylation/activation is likely to protect CRCI in our mouse model.

### Region-specific restoration of PTPRO in the hippocampus of Ptpro^–/–^ mice rescues DOX-induced CRCI.

**Figure 7:**
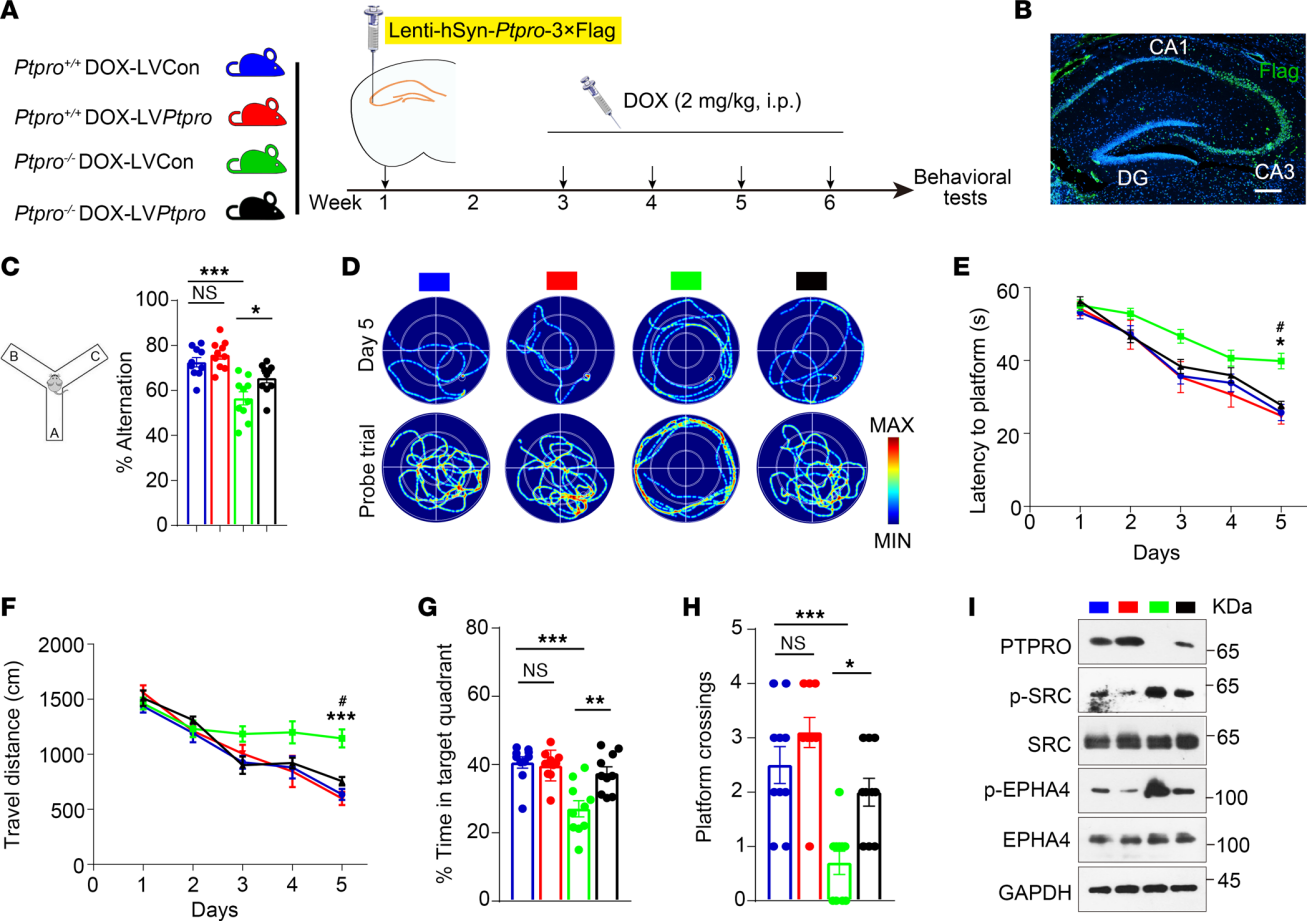
Region-specific restoration of hippocampal PTPRO ameliorates DOX-induced CRCI in *Ptpro^–/–^* female mice. (**A**) Schematic of the experimental design. (**B**) Representative immunofluorescence image of FLAG (green) in the hippocampi of *Ptpro*^–/–^ mice 2 weeks after injection of Lentivirus-hSyn-*Ptpro*-3×Flag into the CA3 region. Scale bar: 200 μm. DG, dentate gyrus. (**C**) Changes in spontaneous alternation behavior in the Y-maze test. (**D**) Representative swimming traces in the MWM test. (**E** and **F**) Training trials were performed in the MWM test, in which the time taken to reach the submerged platform (**E**) and the distances traveled before reaching the submerged platform (**F**) were assesssed, *n* = 10 per group. (**G** and **H**) A probe trial was performed in the MWM test. Shown are the time spent in the target quadrant (**G**) and the number of crossings before reaching the target location (**H**), *n* = 10 per group. (**I**) Immunoblotting of PTPRO, p-SRC, SRC, p-EPHA4, and EPHA4 in the mouse hippocampal CA3 region. Data are representative of 3 independent experiments. Error bars: SEM. NS, not significant; *P < 0.05, **P *<* 0.01, ***P *<* 0.001 by 2-way ANOVA followed by a Tukey-Kramer post hoc test (**C**, **G**, and **H**). *Ptpro^+/+^-LVCon vs. *Ptpro*^–/–^-LVCon; ^#^*Ptpro*^–/–^-LVCon vs. *Ptpro*^–/–^-LV*Ptpro*; *P < 0.05, ***P *<* 0.001; ^#^*P* < 0.05 by 3-way ANOVA followed by a Tukey-Kramer post hoc test (**E** and **F**). All values and statistical analysis of behavioral experiments are provided in Supplemental Table 4.

**Figure 8:**
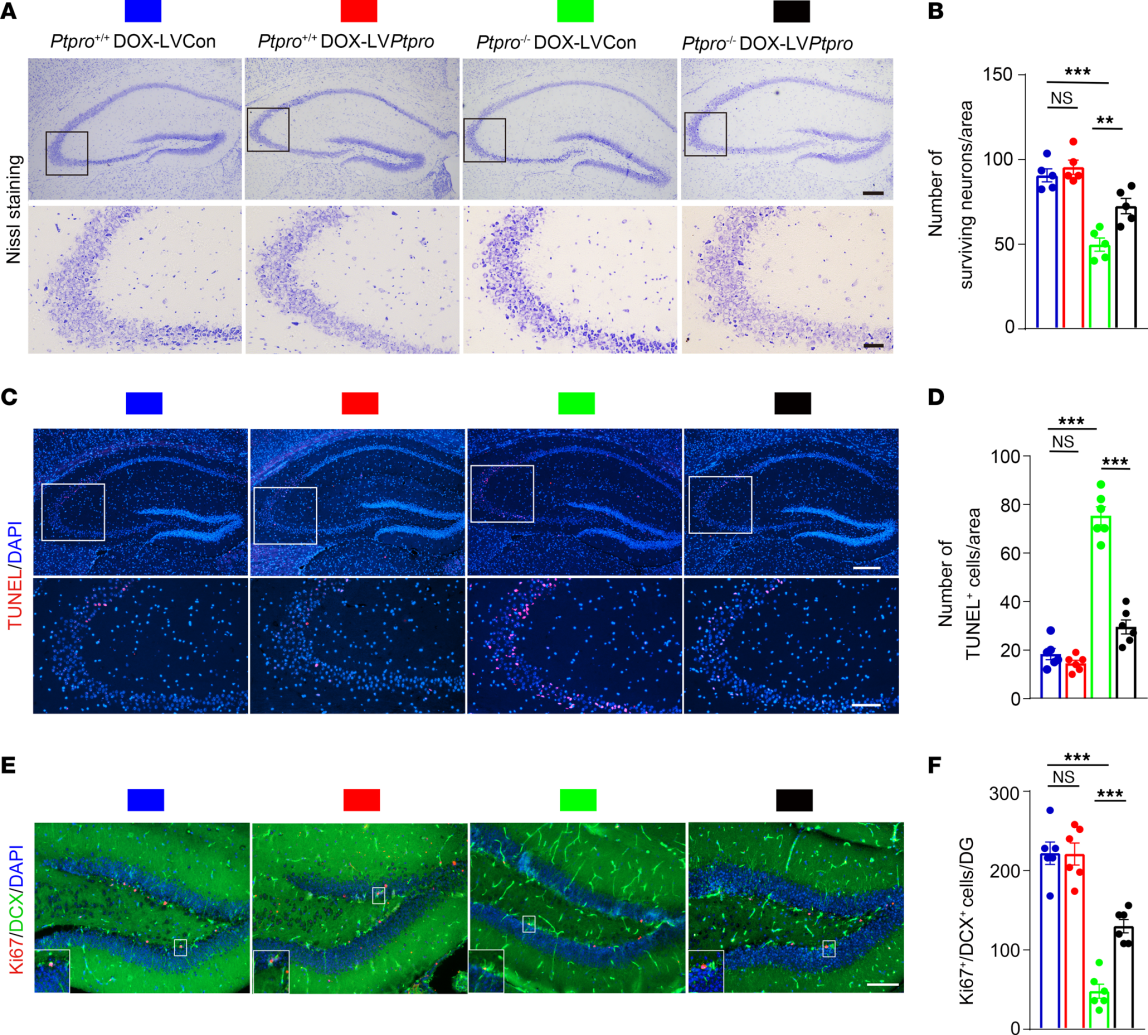
Region-specific restoration of hippocampal PTPRO in *Ptpro^–/–^* female mice treated with DOX prevents neurodegeneration and promotes neurogenesis. (**A**) Nissl staining of the hippocampi (upper panel) and hippocampal CA3 region (bottom panel) in *Ptpro^+/+^*-LVCon, *Ptpro*^+/+^-LV*Ptpro*, *Ptpro*^–/–^-LVCon, and *Ptpro*^–/–^-LV*Ptpro* mice. Scale bars: 200 μm (upper panel), 50 μm (bottom panel). (**B**) Quantification of surviving neurons in the hippocampal CA3 region of *Ptpro^+/+^*-LVCon, *Ptpro*^+/+^-LV*Ptpro*, *Ptpro*^–/–^-LVCon, and *Ptpro*^–/–^-LV*Ptpro* mice. *n* = 5 per genotype. (**C**) TUNEL staining of the hippocampi (upper panel) and hippocampal CA3 region (bottom panel) in *Ptpro^+/+^*-LVCon, *Ptpro*^+/+^-LV*Ptpro*, *Ptpro*^–/–^-LVCon, and *Ptpro*^–/–^-LV*Ptpro* mice. Scale bars: 250 μm (upper panel), 100 μm (bottom panel). (**D**) Quantification of TUNEL-positive hippocampal CA3 cells in *Ptpro^+/+^*-LVCon, *Ptpro*^+/+^-LV*Ptpro*, *Ptpro*^–/–^-LVCon, and *Ptpro*^–/–^-LV*Ptpro* mice. *n* = 6 per genotype. (**E**) Representative images of immature (DCX^+^) and proliferating (Ki67^+^) cells in the hippocampi. Magnified views of areas in the white box are shown in the bottom left corner of each image. Scale bars: 100 μm. (**F**) Quantification of proliferating immature cells in the hippocampi. *n* = 6 per genotype. DG, dentate gyrus. These results are representative of 3 independent experiments. Error bars: SEM. NS, not significant; **P < 0.01, ***P < 0.001 by 1-way ANOVA followed by a Tukey-Kramer post hoc test.

We next asked whether hippocampal PTPRO plays an essential role of protection in CRCI. To assess whether ectopic overexpression of PTPRO specifically in hippocampi can rescue the cognitive dysfunction observed in DOX-induced CRCI, we performed bilateral intrahippocampal injection of control lentivirus (LVCon) or lentivirus expressing *Ptpro* (LV*Ptpro*) into the hippocampal CA3 neurons (PTPRO highly enriched region) of 3-month-old female mice followed by DOX treatment. Two weeks after virus injection, mice were treated with DOX once a week for 4 weeks, and behavioral tests were conducted to evaluate their spatial learning and memory abilities (Figure 7A). Two weeks after virus injection, overexpression of PTPRO in the hippocampus CA3 region was confirmed by checking FLAG expression in hippocampal slices (Figure 7B). Significantly rescued learning and memory abilities of the *Ptpro^–/–^* mice injected with LV*Ptpro* (*Ptpro^–/–^*-LV*Ptpro* mice) were observed both in the Y-maze test (Figure 7C) and in the MWM test (Figure 7, D–H) when compared with *Ptpro*^–/–^ mice injected with LVCon. However, there was no significant change in cognitive ability between *Ptpro^+/+^* mice injected with LV*Ptpro* and LVCon (Figure 7, C–H). In addition, LV-mediated PTPRO restoration led to dephosphorylation/inactivation of SRC and EPHA4 in the hippocampal CA3 region in both *Ptpro*^+/+^ and *Ptpro*^–/–^ mice (Figure 7I). Consistent with the results in Supplemental Figure 5, region-specific restoration of hippocampal PTPRO did not affect blood pressure or CBF and BBB integrity in the CRCI mouse model (Supplemental Figure 11). Furthermore, specific ectopic overexpression of hippocampal PTPRO can effectively rescue neuronal survival, apoptosis, and neurogenesis in *Ptpro*^–/–^, but not *Ptpro*^+/+^ mice (Figure 8).

PTPRO is expressed in both brain and kidney (18). In the kidney, PTPRO regulates the glomerular pressure/filtration rate by affecting podocyte structure and function, and PTPRO reduction is associated with worse outcome of the glomerulus (50). Since impaired kidney function is closely related to cognitive disorders (51), we further examined the relevance of kidney PTPRO to cognitive function in DOX-induced CRCI. We overexpressed PTPRO in the kidney by local injection of LV*Ptpro* in both *Ptpro*^+/+^ and *Ptpro*^–/–^ mice (Supplemental Figure 12A). As shown in Supplemental Figure 12B, PTPRO levels increased in both *Ptpro*^+/+^ and *Ptpro*^–/–^ mice when LV*Ptpro* but not LVCon was injected. However, kidney-expressed PTPRO had no detectable effect on cognitive function (Supplemental Figure 12, C–G). These results provide direct evidence showing that the DOX-induced cognitive dysfunctions in *Ptpro*^–/–^ mice can be largely ameliorated by region-specific restoration of hippocampal but not kidney PTPRO, supporting the essentially protecting role of hippocampal PTPRO in DOX-induced CRCI.

### Region-specific restoration of PTPRO in the hippocampus of Ptpro^–/–^ mice reverses impairment of hippocampal synaptic plasticity in CRCI mice.

**Figure 9:**
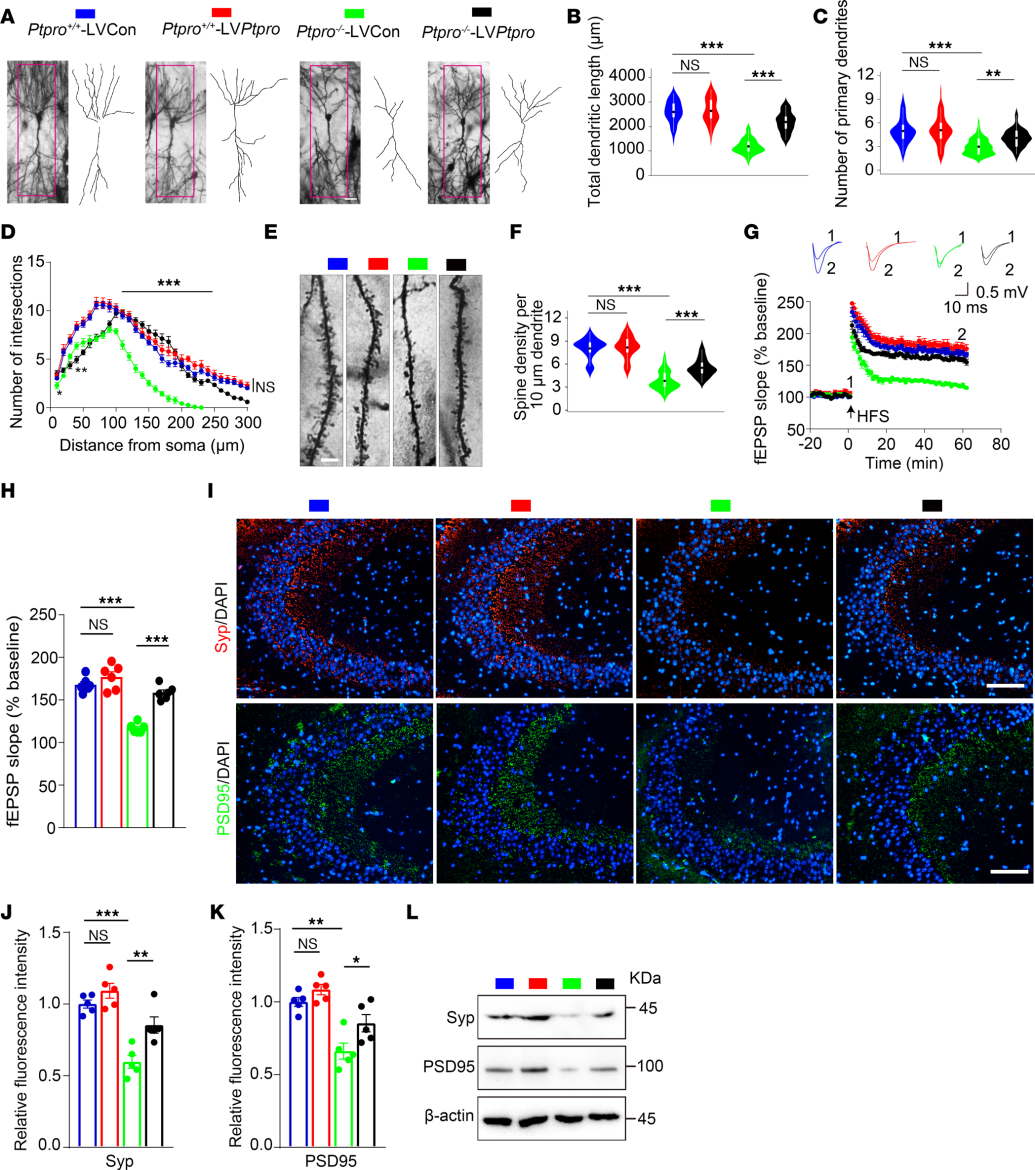
Region-specific restoration of hippocampal PTPRO in *Ptpro*^–/–^ female mice treated with DOX ameliorates synaptic function. (**A**) Representative images and drawings of Golgi-stained hippocampal CA3 pyramidal neurons in each group. Scale bar: 50 μm. (**B** and **C**) Quantification of the total dendritic length and primary dendrites of CA3 pyramidal neurons. *n* = 4 per group. (**D**) Sholl’s analysis of the complexity of CA3 pyramidal neurons. *n* = 4 per group. *Ptpro^–/–^-LVCon vs. *Ptpro*^–/–^-LV*Ptpro*. (**E**) Representative photomicroscopy images of Golgi-stained dendrites of CA3 pyramidal neurons. Scale bars: 5 μm. (**F**) Quantitative analysis of spine densities in CA3 pyramidal neurons. *n* = 6 per group. (**G**) Time course of fEPSP measurements were recorded in the hippocampal CA1 region before and after 100-Hz stimulation in the Schaffer collateral region. Normalized fEPSP slopes were plotted every 1 minute for each group. HFS, high-frequency stimulation. (**H**) The averaged fEPSPs recorded 56–60 minutes after induction of LTP. *n* = 6 slices from 4–6 mice. (**I**) Representative immunofluorescence images of Syp and PSD95 in hippocampal CA3 sections. Scale bars: 200 μm. (**J**) Quantification analysis of the average fluorescence intensity of Syp in hippocampal CA3 sections. *n* = 5 per group. (**K**) Quantification analysis of the average fluorescence intensity of PSD95 in hippocampal CA3 sections. *n* = 5 per group. (**L**) Immunoblotting of Syp and PSD95 in the hippocampi of mice. *n* = 3 per group. These results are representative of 3 independent experiments. Error bars: SEM. NS, not significant; *P < 0.05, **P < 0.01, ***P < 0.001 by 1-way ANOVA followed by a Tukey-Kramer post hoc test (**B**, **C**, **F**, **H**, **J**, and **K**) or 3-way ANOVA followed by Tukey’s multiple-comparison test (**D**).

Golgi staining was performed in *Ptpro^+/+^*-LVCon, *Ptpro^+/+^*-LV*Ptpro*, *Ptpro*^–/–^-LVCon, and *Ptpro*^–/–^-LV*Ptpro* female mice to observe dendrite morphogenesis in the hippocampal CA3 region (Figure 9A). Compared with the CA3 neurons of *Ptpro*^–/–^-LVCon mice, the CA3 neurons in *Ptpro*^–/–^-LV*Ptpro* mice exhibited enhanced dendritic growth and increased primary dendrites (Figure 9, B and C). Sholl’s analysis revealed marked increases in the dendritic branching of CA3 neurons in *Ptpro*^–/–^-LV*Ptpro* mice compared with *Ptpro*^–/–^-LVCon mice (Figure 9D). The spine densities of CA3 pyramidal neurons in *Ptpro*^–/–^-LV*Ptpro* mice increased compared with those of *Ptpro*^–/–^-LVCon mice (Figure 9, E and F). Next, we performed LTP recording to evaluate hippocampal synaptic plasticity. Consistent with behavioral results, the degree of LTP at CA3–CA1 synapses elicited by high-frequency stimulation of Schaffer collaterals was significantly reduced in *Ptpro*^–/–^ mice compared with WT controls following DOX treatment (Figure 9, G and H). LV-mediated PTPRO overexpression rescued impaired LTP in *Ptpro*^–/–^ mice (Figure 9, G and H). Consistently, the relative fluorescence intensities and protein levels of Syp and PSD95 increased in *Ptpro*^–/–^-LV*Ptpro* mice when compared with *Ptpro*^–/–^-LVCon mice (Figure 9, I–L). Consistent with the results in Figures 7 and 8, there was no significant difference in synaptic plasticity between *Ptpro^+/+^* mice injected with LV*Ptpro* or LVCon (Figure 9, A–K). It is likely that the level of hippocampal PTPRO in young mice is sufficiently high to effectively protect DOX-induced CRCI. These results together suggest that hippocampal PTPRO plays essential roles in regulating synaptic plasticity.

### BBR prevents DOX-induced cognitive dysfunction by upregulating hippocampal PTPRO in aged female mice.

**Figure 10:**
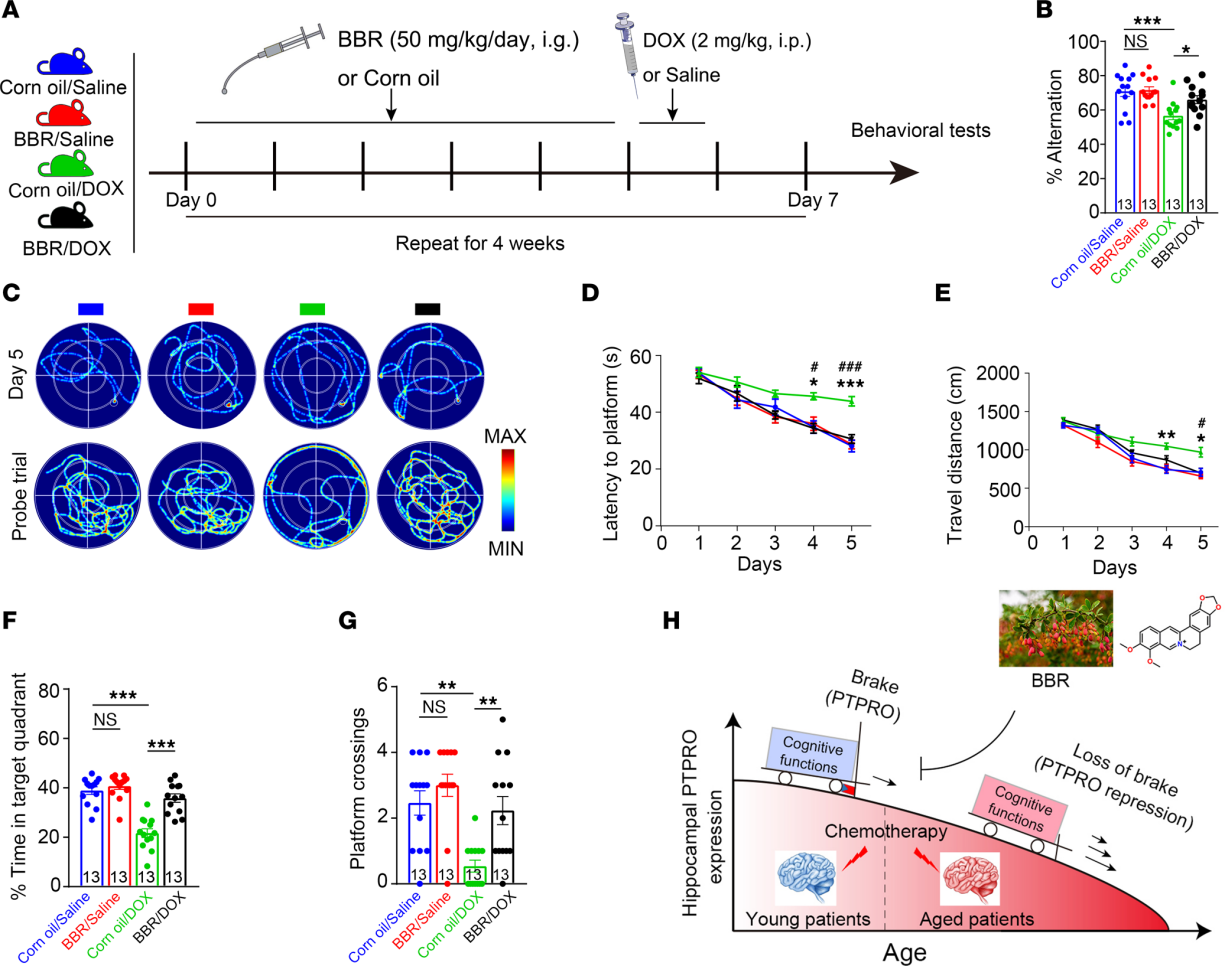
BBR protects against CRCI in aged female mice. (**A**) The scheme of the treatments. (**B**) The changes in spontaneous alternation. *n* =13 per group. (**C**) Representative traces of swimming plot in the MWM test. (**D** and **E**) The time spent to reach the submerged platform (**D**) and the distances spend on the submerged platform (**E**). *Corn oil/saline vs. corn oil/DOX, ^#^corn oil/DOX vs. BBR/DOX; *n* = 13 per group. (**F** and **G**) The time spent in the target quadrant (**F**) and the number of crossings before reaching the target location (**G**), *n* = 13 per group. (**H**) Schematic diagram for explaining the role of PTPRO in CRCI. PTPRO, protein tyrosine phosphatase receptor type O; BBR, berberine. Data are representative of 3 independent experiments. Error bars: SEM. NS, not significant; *P *<* 0.05, **P *<* 0.01, ***P < 0.001; ^#^*P* *<* 0.05, ^###^*P* *<* 0.001 by 2-way ANOVA (**B**, **F**, and **G**) or 3-way ANOVA (**D** and **E**) followed by a Tukey-Kramer post hoc test. All values and statistical analysis of behavioral experiments are provided in Supplemental Table 6.

We next sought to test the interference strategy to experimentally prevent CRCI in the mouse model. The plant alkaloid BBR has been reported for its BBB permeability and neuroprotective effect, as well as its potent modulation of tyrosine kinases (30, 32, 52). Given that CRCI is particularly frequent in elderly cancer patient populations and PTPRO expression in the hippocampus declines with age (Figure 2), the aged WT female mice (18 months old) were pretreated with BBR (or corn oil) for 4 weeks, followed by exposure to DOX (or saline) injection (Figure 10A), and followed by assays of cognitive-behavioral performance. It appeared that BBR had little effect on the behavior of mice when they were not exposed to DOX (Figure 10, B–G). However, BBR effectively reduced DOX-induced cognitive behavioral dysfunctions (Figure 10, B–G, and Supplemental Figure 13, A and B). Consistent with results from both preclinical and clinical studies indicating that BBR can reduce hypertension, protect BBB integrity, and improve CBF (53–55), we found that BBR plays a protective role against DOX-induced BBB damage, systolic and diastolic blood pressure elevation, and CBF reduction (Supplemental Figure 14). These results suggest multiple mechanisms exist in BBR’s protection of DOX-induced cognitive dysfunction in aged mice. Of note, BBR had no influence on the body weight of the animals, suggesting it had no obvious adverse effect on the mice (Supplemental Figure 13C).

Based on their similarly protective effect on DOX-induced dysfunction, we speculated that the protective effects of BBR on cognition may be through the PTPRO signaling pathway. Immunoblotting assays indicate that BBR upregulated PTPRO and downregulated the phosphorylation of SRC and EPHA4 in mouse hippocampi (Supplemental Figure 13D). Furthermore, we also noticed that DOX treatment did not affect the expression of PTPRO but upregulated the levels of phosphorylated SRC and EPHA4 (Supplemental Figure 13D). More interestingly, BBR is capable of counteracting DOX-induced phosphorylation of SRC and EPHA4 (Supplemental Figure 13D). These findings suggest that BBR plays an important neuroprotective role against DOX-induced cognitive dysfunctions in aged mice.

### BBR upregulates hippocampal PTPRO by downregulating miR-25-3p.

**Figure 11:**
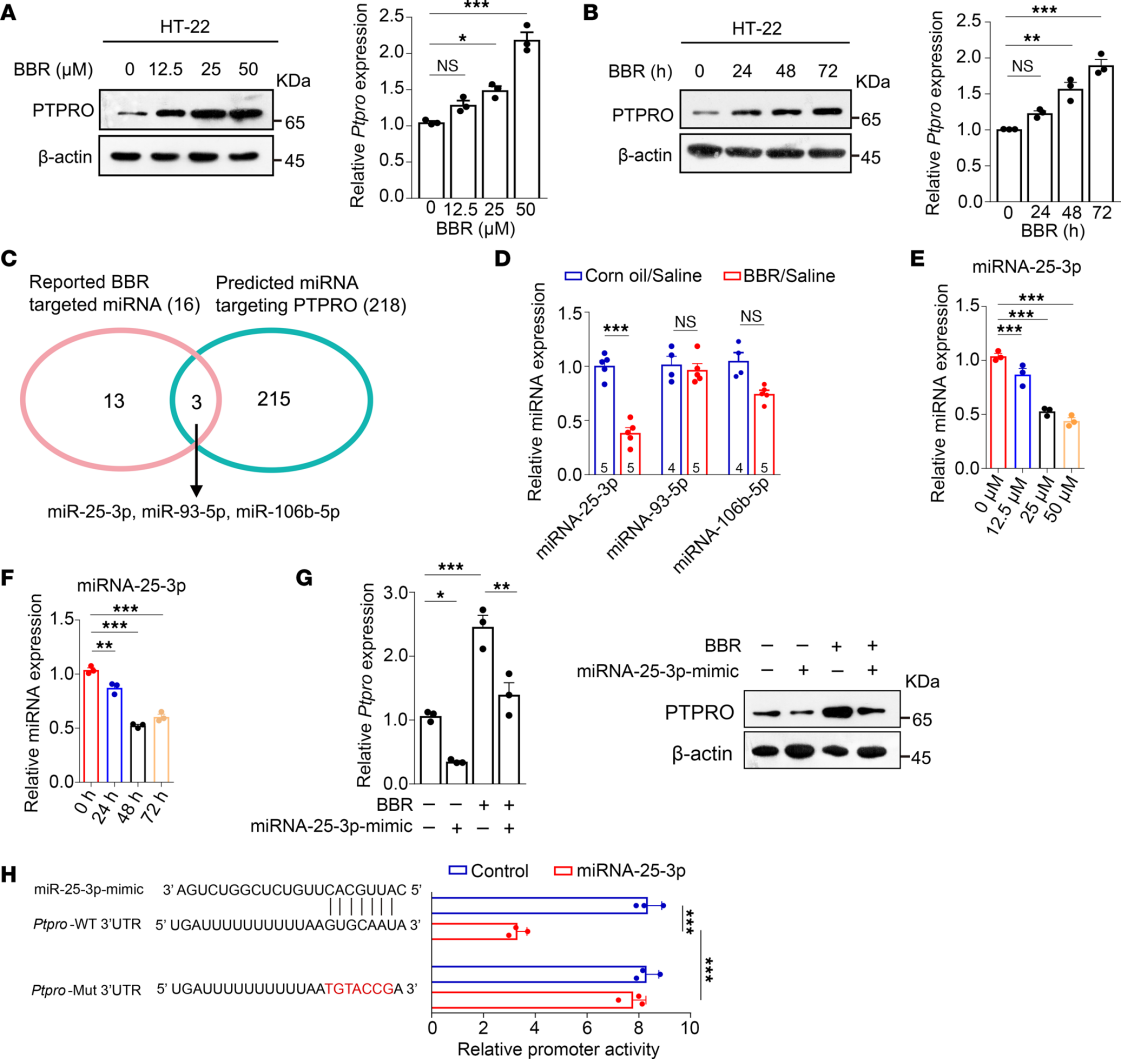
BBR upregulates PTPRO by downregulating miR-25-3p. (**A** and **B**) The protein (left panel) and mRNA (right panel) levels of PTPRO in HT-22 cells treated with different concentrations of BBR for different time periods. (**C**) Venn diagram showing the BBR-regulated miRNAs that potentially target *Ptpro*. (**D**) RT-qPCR analysis of the 3 putative *Ptpro*-targeting miRNAs in hippocampi of mice. *n* = 4–5 per group. (**E** and **F**) RT-qPCR analysis of miR-25-3p expression in HT-22 cells treated with vehicle or with BBR at different concentrations (**E**) or treated with BBR at different time points (**F**). (**G**) HT-22 cells transfected with or without miR-25-3p mimic were treated with or without BBR (25 μM) for 48 hours and then analyzed by RT-qPCR (left panel) and immunoblotting for PTPRO expression (right panel). (**H**) The luciferase reporter plasmid containing WT or mutant *Ptpro* was cotransfected into HT-22 cells with a miR-25-3p mimic. Luciferase activity was determined after 48 hours of transfection. These results are representative of 3 independent experiments. Error bars: SEM. NS, not significant; *P *<* 0.05, **P *<* 0.01, ***P *<* 0.001 by 2-way ANOVA test followed by a Tukey-Kramer post hoc test (**A**, **B**, and **E**–**H**) or 1-way ANOVA followed by a Tukey-Kramer post hoc test (**D**).

Next, we wanted to explore the mechanism in BBR-regulated PTPRO expression in vitro. We found that both protein and mRNA levels of PTPRO were upregulated in dose- and time-dependent manners when the mouse hippocampal cell line HT-22 was treated with BBR (0, 12.5, 25, and 50 μM) (Figure 11, A and B). Multiple lines of evidence implied that BBR could function through targeting different miRNAs (56). We conducted bioinformatics analysis (http://www.targetscan.org/vert72/) and identified 3 BBR-downregulated miRNAs — miR-25-3p, miR-93-5p, and miR-106b-5p — that also potentially interacted with the 3′-UTR of *PTPRO* (Figure 11C and Supplemental Figure 15). To experimentally determine whether any of these miRNAs were involved in BBR-mediated PTPRO upregulation, we estimated the effect of BBR on the levels of these miRNAs. We found that miR-25-3p, but not the other 2 miRNAs, was dramatically downregulated by BBR in time- and dose-dependent manners (Figure 11, D–F). To determine whether miR-25-3p played any role in BBR-upregulated PTPRO, the hippocampal cell line HT-22 transfected with either miR-25-3p mimic or control RNA was treated with BBR (25 μM) or vehicle for 48 hours. The mRNA and protein levels of PTPRO were estimated by RT-qPCR and immunoblotting assays, respectively. Figure 11G shows that miR-25-3p is capable of downregulating not only the mRNA and protein of PTPRO at the basal level but also the BBR-upregulated PTPRO. To demonstrate that miR-25-3p downregulates PTPRO by directly interacting with the 3′-UTR of the *Ptpro* mRNA, we first constructed a luciferase reporter harboring the 3′-UTR of *Ptpro* with either WT or mutant miR-25-3p binding sites and estimated the effect of miR-25-3p using a luciferase assay. When the HT-22 cells were transiently transfected with miR-25-3p mimics with the reporter plasmids, the luciferase activity was only inhibited when the reporter was composed of the WT but not the mutant binding site (Figure 11H). These data suggest that BBR could upregulate PTPRO expression through decreasing miR-25-3p, which directly targets PTPRO.

## Discussion

In view of the fact that CRCI is more frequent in the elderly cancer patient population, CRCI-associated cognitive dysfunction may add to the burden of preexisting age-related performance decline, and therefore the management of older cancer populations is of growing concern. However, there were few studies focusing on older cancer patients with CRCI, aging-associated CRCI model systems, and the underling mechanisms. In this study, we show a substantial enrichment of the tyrosine phosphatase PTPRO in the hippocampus and the age-related decline of hippocampal PTPRO. To establish a CRCI animal model that mimics an elderly cancer patient population with preexisting PTPRO downregulation, *Ptpro*^–/–^ female mice were treated with DOX. *Ptpro* deletion results in severe cognitive phenotypes of CRCI, while site-specific restoration of PTPRO in the hippocampal CA3 region of *Ptpro*^–/–^ female mice significantly reduced CRCI. Furthermore, *Ptpro* deficiency was associated with abnormal activation of hippocampal SRC/EPHA4 when the mice were treated with DOX. The plant-derived BBR can ameliorate CRCI in aged female mice by upregulating hippocampal PTPRO. Mechanistically, BBR upregulates PTPRO by downregulating miR-25-3p and subsequently reducing miR-25-3p–mediated PTPRO degradation.

Compared with most reported mechanistic studies of CRCI using non–genetically modified rodent models treated with chemotherapeutic reagents (46), our study utilized gene-deleted mice to define the underlying genetic factors of CRCI. PTPRO is abundantly expressed in both brain and kidney (18). We found that levels of hippocampal PTPRO are negatively correlated with aging and found no detectable age-dependent change in kidney PTPRO. Thus, we conclude that PTPRO in the hippocampus but not the kidney is an important susceptibility factor to chemotherapy in the elderly. Meanwhile, region-specific restoration of kidney PTPRO in *Ptpro*^–/–^ mice by local infection with lentivirus did not affect cognitive function in DOX-induced CRCI, further indicating that kidney PTPRO is irrelevant to cognition. From this perspective, our conventional knockout mice can be considered as largely equivalent to hippocampal PTPRO knockout, and this notion is further supported by results showing that hippocampal CA3–specific restoration of PTPRO largely rescued the DOX-induced CRCI in *Ptpro*^–/–^ female mice. Moreover, given that CRCI is highly prevalent in women with breast cancer and females are more vulnerable to CRCI (1, 57), we deliberately focused on the ameliorative effects of PTPRO on DOX-induced CRCI in female mice. However, whether PTPRO has similar neuroprotective and neurorestorative effects in male mice needs to be validated further.

Many cancer-related factors and their signaling pathways are deregulated in neurocognitive abnormalities such as AD and dementia (10–13). The tumor suppressor PTPRO is highly enriched in the hippocampi of both humans and mouse, while reduced levels of PTPRO were found in the hippocampi of AD patients (Supplemental Figure 16, A and B). Severe cognitive dysfunctions induced by chemotherapeutic reagent occur in *Ptpro*^–/–^ female mice, and these in vivo data unambiguously demonstrated that the hippocampal PTPRO played an indispensable role in protecting against CRCI. Based on the fact that the PTPRO substrates SRC and EPHA4 are involved in the development of AD, cognitive deficiency, and neuronal differentiation (58–61), it is conceivable that reduced levels of PTPRO lead to phosphorylation/activation of SRC and EPHA4, and therefore the PTPRO/SRC/EPHA4 axis plays what we believe is a previously unrecognized role in CRCI and possibly other cognition-related disorders. Since PTPRO is a tyrosine phosphatase with a broad spectrum of substrates, it would be interesting to test whether other PTPRO-regulated enzymes also participate in PTPRO-mediated functions in CRCI.

There are limited studies on CRCI prevention and/or treatment, particularly those focusing on pathophysiological mechanisms (2, 4, 35, 62). Repurposing existing drugs to prevent CRCI is likely to be cost effective in terms of time and money. We demonstrated here that BBR could effectively alleviate CRCI-related cognitive deficits in aged female mice. We also showed that BBR downregulated miR-25-3p, which directly interacts with and could downregulate PTPRO in vitro. In addition to alleviating the CRCI phenotypes, BBR might modify the trajectory of CRCI at least in a subgroup of elderly cancer patients with preexisting PTPRO downregulation. Given its high permeability across the BBB and tolerability, BBR could be promising as a protective reagent against CRCI in aged patients, and therefore clinical investigation of BBR for CRCI prevention and treatment will be worthwhile.

One of the striking but previously undocumented findings in our study is the age-dependent downregulation of hippocampal PTPRO evidenced by IHC, RT-qPCR, and immunoblotting in mice as well as the results from bioinformatic analyses of age-dependent expression of hippocampal PTPRO in a variety of species. In addition, it has been reported that PTPRO downregulation can be related to viral infections (63), sleep deprivation (64), systemic inflammation (65), alcohol addiction (66), corticosterone levels (67), anxiety (68), unpredictable chronic mild stress (69), prenatal stress (70), and high-fat diet (Supplemental Figure 17). However, whether exposure to these adverse reagents could exacerbate age-dependent hippocampal PTPRO downregulation is unknown. We and others have found that in many cancer types PTPRO downregulation can be partially attributed to promoter methylation (71–79). It would be interesting to determine whether age-dependent downregulation of hippocampal PTPRO is also mediated by promoter hypermethylation. 

In summary, using DOX-treated *Ptpro*^–/–^ female mice to mimic elderly cancer patients with preexisting PTPRO downregulation, we demonstrated that age-decreased PTPRO is an important determining factor of CRCI. In protecting against CRCI, hippocampal PTPRO acts as a “brake” to slow down the deterioration of cognitive function, while the age-associated reduction in hippocampal PTPRO is analogous to loss of the brake and consequently increased susceptibility to CRCI (Figure 10H). On the other hand, BBR ameliorates CRCI-related cognitive dysfunction in aged female mice by upregulating PTPRO (Figure 10H). Therefore, BBR and any reagents possessing similar activities could become promising candidates for CRCI prevention or treatment. Considering the age-related decrease in hippocampal PTPRO, upregulating PTPRO could be a plausible strategy to prevent CRCI in older patients.

## Methods

### Human specimens.

Human specimens were collected from 3 females and 3 males with a median age of 40.5 years (34 to 55) who underwent forensic autopsy between 2012 and 2014 in the Forensic Identification Center of Shantou University (FICSU). Tissue samples included kidney, hippocampus, cerebrum, cerebellum, liver, heart, lung, trachea, testis, ovary, and lymph nodes.

### Animals.

The establishment of *Ptpro*^–/–^ mice on the FVB strain background was described previously (80). All experiments were performed on female FVB mice unless noted otherwise. Young adult mice were between 3 and 4 months old at the time of testing and aged mice were 18 months old. Their genotypes were identified by PCR analyses of tail DNA as described previously with the following primers: *Ptpro*^+/+^ (WT): 5′-AAACCTTAAACTCCTGATCCTCCTGCCTCC-3′ (forward) and 5′-CACTGAATCAAAATGTCCCACCCATGTTTC-3′ (reverse); *Ptpro*^–/–^: 5′-GCCTTCTATCGCCTTCTTGACGAGTTCTTC-3′ (forward) and 5′-CACTGAATCAAAATGTCCCACCCATGTTTC-3′ (reverse). The software PS Power and Sample Size Calculations version 3.0 was used to calculate the sample size (http://biostat.mc.vanderbilt.edu/PowerSampleSize).

### Analysis of human organ-specific transcriptomic data.

Human transcriptomic data from microarray analyses of the following organs — kidney, cerebral cortex, hippocampus, brain, lung, thalamus, colon, spleen, lymph node, retinal, jejunum, spinal cord, epididymis, cerebellum, placenta, pituitary gland, adipose, ileum, duodenum, liver, cervix, stomach, ovary, bone marrow, thymus, heart, bladder, trachea, skeletal muscle, thyroid, adrenal gland, prostate, salivary gland, skin, mammary gland, and testis — were downloaded from the NCBI GEO (http://www.ncbi.nlm.nih.gov/geo/; accession number GSE14938).

### RT-qPCR.

Total RNA was isolated by TRIzol reagent (Thermo Fisher Scientific) according to the manufacturer’s instructions (81). Total RNA (2000 ng) was used for reverse transcription of miRNA and cDNA by All-in-One miRNA First-Strand cDNA Synthesis kit (QP013, GeneCopoeia) and High-Capacity cDNA Reverse Transcription Kit (Applied Biosystems), respectively. Subsequently, miRNA and mRNA expression was quantified with an Applied Biosystems 7500 Real-Time PCR system with an All-in-One miRNA qPCR kit (QP012, GeneCopoeia) and SYBR Green master mix (Applied Biosystems). The qPCR analysis was performed with specific primers for miR-25-3p, miR-93-5p, and miR-106b-5p (GeneCopoeia). *U6* snRNA and *GAPDH* were used for normalization for miRNA and mRNA, respectively. The mRNA primers are as follows: *PTPRO*: 5′-TGGCTGCCAGGAATGTGTTA-3′ (forward) and 5′-TAAGGGGCAGTTCTGTGCTG-3′ (reverse); *Ptpro*: 5′-AAACCTTAAACTCCTGATCCTCCTGCCTCC-3′ (forward) and 5′-CACTGA ATCAAAATGTCCCACCCATGTTTC-3′ (reverse); *GAPDH*: 5′-TGCACCACCAACTGCTTAGC-3′ (forward) and 5′-GGCATGGACTGTGGTCATGAG-3′ (reverse); *U6* snRNA: 5′-CGCTTCGGCAGCACATATAC-3′ (forward) and 5′-TTCACGAATTTGCGTGTCAT-3′ (reverse).

### Surgery and intracranial injection.

Surgeries were carried out as described previously (82). The young mice were anesthetized (Avertin, 13 μL/g, i.p.) and placed in an SA-100 stereotactic instrument (RWD Life Science). A small craniotomy hole was made using a dental drill (OmniDrill35, WPI). A glass cannula filled with virus solution was lowered to the CA3 region (AP, –2.1 mm; ML, ± 2.3 mm; DV, –2.4 mm) and the virus solution (1.0 μL/injection) was injected using a nanoliter injector (NANOLITER 2010, WPI) system at a rate of 0.1 μL per minute sequentially into each side of the hippocampus. VSVG-Lenti-hSyn- *Ptpro-*3×Flag (LV*Ptpro*, viral titer: 1.10 × 10^10^ GC/mL) and VSVG-Lenti-hSyn-EGFP (LVCon, viral titer: 1.37 × 10^9^ GC/mL) were generated and packaged by Shanghai Taitool Bioscience Co., Ltd. The injection cannula was slowly withdrawn 5 minutes after the virus infusion. The scalp was then sealed and injected mice were monitored as they recovered from anesthesia. Behavioral experiments or electrophysiological recordings were performed at least 14 days after virus injection. Virus infection was examined at 2 weeks after virus injection.

### DOX treatment.

DOX treatment dosage and schedule were established in previous studies (83). DOX (2 mg/kg, Sigma-Aldrich) was dissolved in sterile normal saline and injected i.p. once per week for 4 consecutive weeks.

### BBR treatment.

BBR was purchased from MedChemExpress. The BBR dosage was determined based on previous studies (33, 84). The aged mice were randomly divided into 4 groups (*n* = 13 per group): corn oil/saline, BBR/saline, corn oil/DOX, or BBR/DOX. For BBR treatment, the mice were treated with BBR (50 mg/kg in corn oil) by oral gavage, 5 times a week for 4 continuous weeks. At the end of the study, 5 mice of each group were euthanized and the brain tissues were collected for Nissl staining. Three mice of each group were also collected for immunoblotting analysis.

### Y maze.

This test was performed as described previously (85). The Y maze was a 3-arm (each 30 cm long, 8 cm wide, and 15 cm in height) maze with equal angles between all arms. The 3 identical arms were randomly designated as start arm, novel arm, and another arm. The percentage of triads in which all 3 arms are represented was recorded as an alternation to estimate short-term memory of the last arms entered. An alternation is defined as a visit to all 3 arms without reentry (ABC, ACB, BAC, BCA, CAB, or CBA). The total number of arm entries was used as a measure for locomotor activity, while the spontaneous alternation percentage (SAP) was used as a measure of spatial working memory. To calculate the SAP, the total number of alternations (i.e., every time a mouse explored the 3 arms consecutively) was divided by the total possible alternations (i.e., the number of arm entries minus 2) and multiplied by 100.

### MWM.

Spatial memory abilities of mice were examined in the MWM. The test was conducted in a circular tank (150 cm in diameter and 50 cm in depth) with a 10-cm diameter central round platform hidden 1 cm below the surface of the water that was maintained at 24°C. The pool was divided arbitrarily into 4 quadrants labeled N-S-E-W. Each mouse was given 4 swimming trials per day for 5 days. The start position was randomized among the 4 quadrants (N-S-E-W) for each trial. Each trial lasted until the animal found the platform or for a maximal observation period of 60 seconds, and the animals that failed to find the platform within 60 seconds were guided by the experimenter to the platform. Mice remained on the platform for 10 seconds before being removed to the home cage. On the sixth day, a probe trial without the platform was performed in order to measure the retention of spatial memory. For each trial, the time required to locate the hidden platform (escape latency), distance traveled (path length), percentage time in quadrant, and number of crosses were recorded using an EthoVision video tracking system.

### Tissue staining.

IHC staining was performed as previously described (81). In brief, brain samples were fixed in 10% formalin with phosphate buffer (pH 7.4) and subsequently embedded in paraffin. The paraffin-embedded tissues were cut into 4-μm sections and mounted on glass slides. Thereafter, tissues were dewaxed and subsequently rehydrated. Antigen retrieval was performed by soaking in 10 mM Tris/1 mM EDTA, pH 9.0 and microwaved on medium power (400 W) for 25 minutes, and then the sections were rinsed in Tris-buffered saline. Endogenous peroxidase activity was blocked using 3% H_2_O_2_ for 15 minutes. Sections were then incubated with a primary antibody against one of the following antigens: PTPRO (1:200; catalog 12161-1-AP, Proteintech Group), p-SRC (1:100; catalog 2101, Cell Signaling Technology), SRC (1:100; catalog sc-8056, Santa Cruz Biotechnology), p-EPHB2 (1:400; catalog ab61791, Abcam), EPHB2 (1:200; catalog 83029, Cell Signaling Technology), p-EPHA4 (1:100; catalog EP2731, ECM Biosciences), and EPHA4 (1:200; catalog sc-365503, Santa Cruz Biotechnology). The sections were then incubated with the appropriate HRP-conjugated secondary antibodies at 37°C for 1 hour. The color was developed by incubation with 3,3′-diaminobenzidine (DAB) substrate. The nuclei were counterstained with hematoxylin. For immunofluorescent staining, sections were incubated with antibodies against the following proteins: Ki67 (1:400; catalog 9129, Cell Signaling Technology), DCX (1:200; catalog sc-217390, Santa Cruz Biotechnology), PSD95 (1:200; catalog 3450, Cell Signaling Technology), and Syp (1:250; catalog ab32127, Abcam). Appropriate secondary antibodies (Alexa Fluor 488 or 594; Invitrogen) were used followed by incubation with DAPI. A total of 6 sections per brain containing the hippocampus and 5 to 6 mice per group were stained with antibodies as mentioned above. Images were digitally captured using a Leica DMi8 fluorescence microscope (Leica Microsystems). Cell counting and staining intensity were quantified using Fiji software (https://imagej.net/software/fiji/).

### Golgi staining.

For Golgi-Cox impregnation of neurons, the FD Rapid GolgiStain kit (FD NeuroTechnologies) was used according to the manufacturer’s protocol. The brains were cut in sections of 150 μm thickness using a vibratome. Hippocampal sections were collected on a 0.3% gelatin solution, dried at room temperature, dehydrated in alcohol, and cleared with xylene. Finally, they were mounted on 0.3% gelatinized slides. Bright-field images were taken on a Cytation 5 multi-mode plate reader (BioTek). Dendrites were traced, and their lengths were measured using the Fiji plugin Simple Neurite Tracer. For Sholl’s analysis, we used NeuronStudio to plot proximal complexity and branching of apical and basal dendritic domains in hippocampal CA3 pyramidal neurons.

### Immunoblotting.

Immunoblotting was performed as described previously (80, 81). Briefly, the CA3 tissues were homogenized and proteins were extracted using RIPA lysis buffer (Millipore). Protein concentrations were quantified by the BCA method. Protein samples were resolved by SDS–polyacrylamide gel electrophoresis and then transferred to polyvinylidene difluoride membrane (Millipore). The membranes were immersed in blocking buffer (5% skim milk in PBS) for 1 hour at room temperature and incubated overnight with primary antibodies used against the following proteins: PTPRO (1:1000; catalog 12161-1-AP, Proteintech Group), p-SRC (1:1000; catalog 2101, Cell Signaling Technology), SRC (1:1000; catalog sc-8056, Santa Cruz Biotechnology), p-EPHB2 (1:1000; catalog ab61791, Abcam), EPHB2 (1:1000; catalog 83029, Cell Signaling Technology), p-EPHA4 (1:1000; catalog EP2731, ECM Biosciences), EPHA4 (1:1000; catalog sc-365503, Santa Cruz Biotechnology), PSD95 (1:1000; catalog 3450, Cell Signaling Technology), Syp (1:1000; catalog ab32127, Abcam), β-actin (1:1000; catalog 4967, Cell Signaling Technology), and GAPDH (1:1000; catalog ab8245, Abcam). After incubation with the primary antibodies, the secondary antibodies were added and incubated for 2 hours at room temperature. Immunoreactive bands were visualized by enhanced chemiluminescence (ECL, Pierce). Relative protein levels were quantified by Fiji software and normalized to that of GAPDH.

### Hippocampal slice electrophysiology.

All animals were anesthetized (Avertin, 13 μL/g, i.p.) and were euthanized by decapitation. Coronal slices of the hippocampus (350 μm) were cut using a vibratome (VT1200S, Leica Microsystems) in ice-cold artificial cerebrospinal fluid (ACSF, in mM: 119 NaCl; 2.5 KCl, 1 NaH_2_PO_4_, 11 glucose, 26.2 NaHCO_3_, 2.5 CaCl_2_, 1.3 MgCl_2_, and 290 mOsm, at pH 7.4), which was saturated with 95% O_2_ and 5% CO_2_. Recordings began after at least 30 minutes of incubation. To record the extracellular field excitatory postsynaptic potentials (fEPSPs), a glass micro-electrode (4–8 MΩ, filled with ACSF) was placed in the stratum radiatum of the CA1 region, and a bipolar tungsten stimulating electrode was placed along the Schaffer collateral fibres 100–150 μm away from the recording pipette. The intensity of the stimulation was adjusted to produce a fEPSP with an amplitude of 30%–40% of the maximal response. After a stable baseline was established, LTP was induced by applying 4 trains (1 second at 100 Hz) spaced 20 seconds, and potentiation was measured for 1 hour after LTP induction at 0.033 Hz. Data were collected and digitized by MultiClamp 700B (Axon Instruments). For each experiment, fEPSP slopes are expressed as a percentage of average pretetanus baseline slope values.

### Cell culture and treatment.

HT-22 cells, a mouse hippocampal neuronal cell line, were supplied by FuHeng Cell Center. Cells were cultured in DMEM (Sigma-Aldrich) supplemented with 10% FBS in 10-cm dishes and incubated at 37°C in an atmosphere containing 5% CO_2_. Cells were confirmed to be mycoplasma negative. Cells at 60% confluence were treated with BBR (MedChemExpress). BBR was dissolved in dimethyl sulfoxide (DMSO), which was used as the vehicle control. Prior to treatment with the various compounds, the medium was exchanged with Eagle’s MEM (containing sodium pyruvate and vitamins) that did not contain serum. After coincubation wtih BBR, the whole-cell lysates were prepared from treated cells for immunoblotting and RT-qPCR, respectively.

### Statistics.

All analyses were conducted using SPSS statistics (version 23.0, IBM Corp.) and Prism (version 7.0b, GraphPad Software). Summary statistics reporting means, SEM, and 95% CIs are stated as appropriate. Comparisons between independent groups were performed with Student’s *t* test or 1-way ANOVA with post hoc intergroup comparisons, where appropriate, if the sample passed the test for normal distribution (Shapiro-Wilk). Otherwise, a rank-sum test was used. All statistical analyses of behavioral data were conducted using either 2-way ANOVA or 3-way ANOVA, where appropriate. Post hoc planned comparisons were applied for significant effects and interactions. The sample sizes (*n*) are provided in the figures and figure legends. For detailed information and numerical statistical results, see Supplemental Tables 1, 2, and 4–6. *P* values less than 0.05 were considered to be significant, and all tests were 2-sided.

### Study approval.

The study involved human samples was approved by the Ethics Committee for the use of human subjects of Shantou University Medical College (approval no. 04-070, Shantou, China). The study was conducted in accordance with the principles of the Declaration of Helsinki. All the animal experiments were approved by the Animal Care and Use Committee of Jinan University (approval no. IACUC-20190711-07).

## Author contributions

ZY, HD, Y Luo, JZ, and LD contributed equally to this work. HZ conceived and supervised the research. ZY, JZ, KZ, LD, Y Lin, XH, and QL contributed to the mouse model constructions and performed mouse behavioral experiments. ZY, JZ, and LW performed electrophysiological experiments in the hippocampus and associated data analysis. ZY, JZ, Y Luo, SL, XY, and SW performed immunohistochemical analyses of human and mouse tissues, cell biological experiments, and dendritic spine analysis in hippocampi. HD, WW, and QH helped to perform behavioral experiments and associated data analysis. HD, HX, WZ, XQ, and YP helped to perform immunohistochemical analyses and provided technical support. HZ, SCJY, DZ, HD, and ZY wrote the manuscript and gave scientific advice.

## Supplementary Material

Supplemental data

## Version 1 07/24/2023

Electronic publication
