# Supplementary material for: Age-related decline in hippocampal tyrosine phosphatase PTPRO is a mechanistic factor in chemotherapy-related cognitive impairment
Source: JCI Insight. 2023 Jul 24;8(14):e166306. doi: 10.1172/jci.insight.166306 (PMC10443805; doi:10.1172/jci.insight.166306)
Supplement: Supplemental data [file jciinsight-8-166306-s103.pdf]

Supporting Information

Age-related decline in hippocampal tyrosine phosphatase PTPRO is a mechanistic factor in chemotherapy-related cognitive impairment

Zhimeng Yao[1,2,3#], Hongmei Dong[3#], Yichen Luo[3#], Jianlin Zhu[2,3#], Liang Du[3#], Qing Liu[4], Shixin Liu[5], Yusheng Lin[3,6,7], Lu Wang[3], Shuhong Wang[3], Wei Wei[8], Keke Zhang[8], Qingjun Huang[9], Xiaojun Yu[10], Weijiang Zhao[11,12], Haiyun Xu[9,13], Xiaofu Qiu[14], Yunlong Pan[2,15], Xingxu Huang[16], Sai-Ching Jim Yeung[17], Dianzheng Zhang[18], Hao Zhang[2,19*]

Author affiliations

[1]Department of Urology Surgery, The First Affiliated Hospital of Jinan University, Jinan University, Guangzhou, Guangdong, China

[2]Department of General Surgery, The First Affiliated Hospital of Jinan University, Jinan University, Guangzhou, Guangdong, China

[3]Institute of Precision Cancer Medicine and Pathology, School of Medicine, Jinan University, Guangzhou, Guangdong, China

[4]Department of Pathology, The First People's Hospital of Foshan, Foshan, Guangdong, China

[5]Department of Thoracic Surgery, The First Affiliated Hospital of Jinan University, Jinan University, Guangzhou, Guangdong, China

[6]Graduate School, Shantou University Medical College, Shantou, Guangdong, China

[7]Department of Hematology, University Medical Center Groningen, University of Groningen, Groningen, the Netherlands

[8]Department of Pathophysiology, Key Laboratory of State Administration of Traditional Chinese Medicine of the People's Republic of China, School of Medicine, Jinan University, Guangzhou, Guangdong, China

[9]Shantou University Mental Health Center, Shantou University Medical College, Shantou, Guangdong, China

[10]National Key Disciplines, Department of Forensic and Pathology, Shantou University Medical College, Shantou, Guangdong, China

[11]Center for Neuroscience, Shantou University Medical College, Shantou, Guangdong, China

[12]Cell Biology Department, Wuxi School of Medicine, Jiangnan University, Wuxi, Jiangsu, China

[13]The Affiliated Kangning Hospital, Wenzhou Medical University, Wenzhou, Zhejiang, China

[14]Department of Urology, Guangdong Second Provincial General Hospital, Guangzhou, Guangdong, China

[15]Minister of Education Key Laboratory of Tumor Molecular Biology, Jinan University, Guangzhou, Guangdong, China

[16]Gene Editing Center, School of Life Sciences and Technology, ShanghaiTech University, Shanghai, China

[17]Department of Emergency Medicine, University of Texas MD Anderson Cancer Center and Department of Endocrine Neoplasia and Hormonal Disorders, University of Texas MD Anderson Cancer Center, Houston, USA

[18]Department of Biomedical Sciences, Philadelphia College of Osteopathic Medicine, 4170 City Ave, Philadelphia, USA

[19]Institute of Precision Cancer Medicine and Pathology, School of Medicine, and Minister of Education Key Laboratory of Tumor Molecular Biology, Jinan University, Guangzhou, Guangdong, China

[#]These authors have contributed equally to this work.

Running title: Age-related decline of PTPRO and chemobrain

Conflict of Interest： The authors have declared that no conflict of interest exists.

Supplementary materials and methods

TUNEL staining and Nissl staining

Apoptosis examination was performed using ApopTag plus peroxidase *in situ* apoptosis detection kit following the manufacturer's instruction. Nissl staining was performed using Nissl staining solution (C0117, Beyotime Biotechnology), according to the manufacturer's instructions. Stained sections were visualized under a Leica DMi8 microscopy (Leica Microsystems). The number of TUNEL-positive cells or survival neurons in CA3 of the hippocampal was counted using Fiji software.

Cell counts

Five to six animals of each genotype and treatment were used. Quantification was done in every fourth, fifth and sixth section, apart from by at least 150 µm, in one of six series, with a total of 6 sections/mice analyzed as described (1, 2). The CA3 region and DG of the hippocampus were identified at low magnification and $Ki67^{+}/DCX^{+}$ cells from every fourth section, Nissl-positive cells from every fifth section and TUNEL-positive cells from every sixth section covering the whole dorsal hippocampus were counted for multiple high-power microscopic fields and averaged. The number of labeled cells was determined by bilaterally counting cells in a total of six sections and averaged for each mouse. The total number of $Ki67^{+}/DCX^{+}$ cells was determined by multiplying the total number of labeled cells from all six sections by section periodicity (i.e., 6).

Luciferase assay

HT-22 cells were transiently co-transfected with miRNA mimics and WT or mutated *Ptpro* 3'-UTR gaussian luciferase reporter constructs. After 48 hours, the medium was collected for measurement of the luciferase activities using the Secrete-Pair™ Dual Luminescence Assay Kit (GeneCopoeia, USA) according to the manufacturer's instructions.

Gene set enrichment analyses

The GEO dataset (GSE29378, GSE90696, GSE111494 and GSE60911) were obtained from the Gene Expression Omnibus and analyzed using GSEA software as previously described (http://www.software. broadinstitute.org/gsea/index.jsp).

Blood pressure monitoring

Blood pressure was measured by the non-invasive computerized tail-cuff blood pressure system (CODA-HT8; Kent Scientific) at the experimental endpoints as described previously (3). Before the experiment, the mice were warmed with a heating pad for 5 minutes. Blood pressure was continuously recorded always from 7.00 to 9.00 a.m. A total of 7-10 readings were taken for each mouse, at 1minute intervals.

Cerebral blood flow (CBF) measurement

CBF was assessed by the laser Doppler flowmeter (moor FLPI-2, Moor Instruments). Briefly, the mice were anesthetized and the fur on the head was removed by shaving. A midline skin incision was made at the back of the brain to expose the skull. Then, the skull was placed 10 cm below the scan head to acquired and analysed CBF perfusion in real time.

Evans blue extravasation

Evans blue extravasation was conducted to evaluate blood brain barrier (BBB) integrity of mice in each group. In brief, evans blue dye (4 mL/kg, Sigma-Aldrich) in was injected into the tail vein and the mice were sacrificed 1 hour later. To qualify the extravasated Evans blue, the mouse brains were homogenized in 1 mL of PBS and then centrifuged (1000 ×g) for 15 minutes to collect the supernatant. the supernatant were then homogenized by 50% trichloroacetic acid at 4℃ for 24 hours. The mixture was centrifuged at 2000 ×g for 15 minutes at 4℃, and the supernatant was collected. Optical density of the supernatant was measured using Cytation5 multi-mode plate reader (BioTek, USA) at 620 nm.

Orthotopic transplantation experiments

For orthotopic transplantation experiments, primary PyMT tumor pieces were isolated from PyMT tumors (11-week-old FVB mice). 6-8 week old host FVB mice (*Ptpro*$^{+/+}$ or *Ptpro*$^{-/-}$) were anesthetized and then made a 4 mm incision under the nipple of the right abdominal mammary gland to create a pocket where a tumor piece (2×2 mm) was inserted as previously described (4). Tumor growth was assessed twice weekly with a caliper and tumor volume was calculated using length×width^2×(π/6). Mice were given DOX or saline when tumor volume reached approximately 80 mm^3.

Microinjection of *Ptpro*-overexpressing lentivirus into mouse kidney

The 8-week-old mice *Ptpro*$^{+/+}$ and *Ptpro*$^{-/-}$ mice were treated with lentivirus for overexpression of *Ptpro* gene. The lentivirus were generated and packaged by Shanghai Taitool Bioscience Co., Ltd. (China). According to the manufacturer's instruction, the mice administered in situ renal injection with LV-*Ptpro* (virus titers: 2.0×10^9 GC/mL) or LV-Con (virus titers: 1.7×10^9 GC/mL) at 300 μL volumes of vector per mouse. To deliver the lentivirus vector, we performed multi-point injection by using Hamilton syringes at 6 distributed points around the renal cortex region (shown in Supplemental Figure 12A) as described previously (5).

References

1. Bozzi Y, et al. Neuroprotective role of dopamine against hippocampal cell death. *J Neurosci.* 2000; 20(22):8643-8649.
2. Zheng J, et al. Adult Hippocampal Neurogenesis along the Dorsoventral Axis Contributes Differentially to Environmental Enrichment Combined with Voluntary Exercise in Alleviating Chronic Inflammatory Pain in Mice. *J Neurosci.* 2017; 37(15):4145-4157.
3. Wang Y, et al. Measuring Blood Pressure Using a Noninvasive Tail Cuff Method in Mice. *Methods in molecular biology.* 2017; 1614:69-73.
4. Roswall P, et al. Microenvironmental control of breast cancer subtype elicited through paracrine platelet-derived growth factor-CC signaling. *Nature medicine.* 2018; 24(4):463-473.
5. Li J, et al. B7-1 mediates podocyte injury and glomerulosclerosis through communication with Hsp90ab1-LRP5-beta-catenin pathway. *Cell death and differentiation.* 2022; 29(12):2399-2416.

Supplementary Tables

Table S1. Statistical analysis of behavioral studies in *Ptpro*$^{+/+}$ and *Ptpro*$^{-/-}$ female mice after administration of DOX or saline.

Parameter	Comparison	Results
Y maze (*n* = 15)	DOX, and genotype	two-way ANOVA; main effect of DOX: $F_{(1, 56)}$ = 44.92, $P < 0.0001$; main effect of genotype: $F_{(1, 56)}$ = 16.09, $P = 0.0002$; DOX×genotype interaction: $F_{(1, 56)}$ = 6.143, $P = 0.0162$
Morris water maze-initial training (*n* = 13)		
Latency to platform	DOX, genotype, and time	three-way ANOVA; main effect of DOX: $F_{(1, 240)}$ = 43.87, $P < 0.0001$; main effect of genotype: $F_{(1, 240)}$ = 12.81, $P = 0.0004$; main effect of time: $F_{(4, 240)}$ = 50.53, $P < 0.0001$; time×DOX interaction: $F_{(4, 240)}$ = 4.255, $P = 0.0024$; time×genotype interaction: $F_{(4, 240)}$ = 1.413, $P = 0.2302$; DOX × genotype interaction: $F_{(1, 240)}$ = 8.726, $P = 0.0034$; DOX×genotype×time interaction: $F_{(4, 240)}$ = 0.7605, P = 0.5519
Distance traveled to platform	DOX, genotype, and time	three-way ANOVA; main effect of DOX: $F_{(1, 240)}$ = 110.9, $P < 0.0001$; main effect of genotype: $F_{(1, 240)}$ = 24.63, $P < 0.0001$; main effect of time: $F_{(4, 240)}$ = 107.8, $P < 0.0001$; time×DOX interaction: $F_{(4, 240)}$ = 7.926, $P < 0.0001$; time×genotype interaction: $F_{(4, 240)}$ = 3.019, $P = 0.0186$; DOX × genotype interaction: $F_{(1, 240)}$ = 17.43, $P < 0.0001$; DOX×genotype×time interaction: $F_{(4, 240)}$ = 3.684, P = 0.0062
Morris water maze-probe trial (*n* = 13)		
Percentage time in quadrant	DOX, and genotype	two-way ANOVA; main effect of DOX: $F_{(1, 48)}$ = 164.4, $P < 0.0001$; main effect of genotype: $F_{(1, 48)}$ = 48.77, $P < 0.0001$; DOX×genotype interaction: $F_{(1,48)}$ = 11.41, $P = 0.0015$
Number of platform crossings	DOX, and genotype	two-way ANOVA; main effect of DOX: $F_{(1,48)}$ = 103.5, $P < 0.0001$; main effect of genotype: $F_{(1, 48)}$ = 12.03, $P = 0.0011$; DOX×genotype interaction: $F_{(1, 48)}$ = 1.924, $P = 0.1718$

Table S2. Statistical analysis of behavioral studies in *Ptpro*$^{+/+}$ and *Ptpro*$^{-/-}$ female mice after administration of DOX or saline in the MMTV-PyMT breast cancer model.

Parameter	Comparison	Results
Morris water maze-initial training (n = 8)		
Latency to platform	DOX, genotype, and time	three-way ANOVA; main effect of DOX: $F_{(1, 140)}$ = 118.6, P < 0.0001; main effect of genotype: $F_{(1, 140)}$ = 15.31, P = 0.0001; main effect of time: $F_{(4, 140)}$ = 108.5, P < 0.0001; time×DOX interaction: $F_{(4, 140)}$ = 11.11, P < 0.0001; time×genotype interaction: $F_{(4, 140)}$ = 3.779, P = 0.0060; DOX × genotype interaction: $F_{(1, 140)}$ = 5.162, P = 0.0246; DOX×genotype×time interaction: $F_{(4, 140)}$ = 1.082, P = 0.3676
Distance traveled to platform	DOX, genotype, and time	three-way ANOVA; main effect of DOX: $F_{(1, 140)}$ = 91.68, P < 0.0001; main effect of genotype: $F_{(1, 140)}$ = 17.84, P < 0.0001; main effect of time: $F_{(4, 140)}$ = 117.9, P < 0.0001; time×DOX interaction: $F_{(4, 140)}$ = 10.71, P < 0.0001; time×genotype interaction: $F_{(4, 140)}$ = 3.725, P = 0.0065; DOX × genotype interaction: $F_{(1, 140)}$ = 8.245, P = 0.0047; DOX×genotype×time interaction: $F_{(4, 140)}$ = 1.415, P = 0.2320
Morris water maze-probe trial (n = 8)		
Percentage time in quadrant	DOX, and genotype	two-way ANOVA; main effect of DOX: $F_{(1, 28)}$ = 149.9, P < 0.0001; main effect of genotype: $F_{(1, 28)}$ = 18.92, P = 0.0002; DOX×genotype interaction: $F_{(1, 28)}$ = 6.202, P = 0.0190
Number of platform crossings	DOX, and genotype	two-way ANOVA; main effect of DOX: $F_{(1,28)}$ = 62.87, P < 0.0001; main effect of genotype: $F_{(1, 28)}$ = 7.916, P = 0.0089; DOX×genotype interaction: $F_{(1, 28)}$ = 3.206, P = 0.0842

Table S3. List of reported PTPRO substrates, cognitive-related tyrosine kinases and neurotoxic-related tyrosine kinases.

Reported PTPRO substrates	Cognitive-related tyrosine kinases	Neurotoxic-related tyrosine kinases
EGFR	*AATK*	*ABl1*
EPHA2	*ABL1*	*ABL2*
EPHA4	*AXL*	*ALK*
EPHB2	*CLK1*	*AXL*
ERBB2	*CSK*	*BTK*
NPCD	*DDR1*	*CSK*
SRC	*EGFR*	*DYRK2*
VCP	*EPHA4*	*EPHA2*
	EPHA7	*EPHA3*
	EPHB2	*EPHA4*
	ERBB3	*EPHA5*
	ERBB4	*EPHA7*
	FGFR1	*EPHA8*
	FGFR2	*EPHB1*
	FGFR3	*EPHB2*
	FYN	*EPHB3*
	GAK	*ERBB2*
	GSG2	*ERBB3*
	IGF1R	*ERBB4*
	INSR	*FER*
	KIT	*FES*
	MATK	*FGFR1*
	MELK	*FGFR2*
	MET	*FGFR3*
	MUSK	*FYN*
	NTRK1	*GAK*
	NTRK2	*KDR*
	NTRK3	*KIT*
	PDGFRB	*LTK*
	PIK3R4	*LYN*
	PKDCC	*MET*
	PLK4	*NTRK1*
	PTK2B	*NTRK2*
	ROS1	*NTRK3*
	RYK	*PDGFRA*
	SRC	*PDGFRB*
	TESK1	*PTK2*
	TTK	*PTK2B*
	TWF1	*PTK6*
	TYK2	*PTK7*
		RET
		RYK
		SRC
		TWF2

Table S4. Statistical analysis of behavioral studies in CRCI female mice infused with Lentivirus.

Parameter	Comparison	Results
Y maze (*n* = 10)	Virus and genotype	two-way ANOVA; main effect of genotype: $F_{(1, 36)}$ = 29.64, $P < 0.0001$; main effect of virus: $F_{(1, 36)}$ = 5.641, $P = 0.0230$; genotype × virus interaction: $F_{(1, 36)}$ = 2.349, $P = 0.1341$
Morris water maze-initial training (*n* = 10)		
Latency to platform	Virus, genotype, and time	three-way ANOVA; main effect of genotype: $F_{(1, 180)}$ = 24.39, $P < 0.0001$; main effect of virus: $F_{(1, 180)}$ = 10.3, $P = 0.0016$; main effect of time: $F_{(4, 180)}$ = 74.17, $P < 0.0001$; time × genotype interaction: $F_{(4, 180)}$ = 1.44, $P = 0.2228$; time × virus interaction: $F_{(4, 180)}$ = 1.38, $P = 0.2426$; genotype × virus interaction: $F_{(1, 180)}$ = 6.403, $P = 0.0122$; genotype × virus × time interaction: $F_{(4, 180)}$ = 0.9693, $P = 0.4257$
Distance traveled to platform	Virus, genotype, and time	three-way ANOVA; main effect of genotype: $F_{(1, 180)}$ = 23.47, $P < 0.0001$; main effect of virus: $F_{(1, 180)}$ = 7.649, $P = 0.0063$; main effect of time: $F_{(4, 180)}$ = 55.64, $P < 0.0001$; time × genotype interaction: $F_{(4, 180)}$ = 2.943, $P = 0.0218$; time × virus interaction: $F_{(4, 180)}$ = 2.736, $P = 0.0303$; genotype × virus interaction: $F_{(1, 180)}$ =6.251, $P = 0.0133$; genotype × virus × time interaction: $F_{(4, 180)}$ = 2.326, $P = 0.0582$
Morris water maze-probe trial (*n* =10)		
Percentage time in quadrant	Virus and genotype	two-way ANOVA; main effect of genotype: $F_{(1, 36)}$ = 18.02, $P = 0.0001$; main effect of virus: $F_{(1, 36)}$ = 6.579, $P = 0.0146$; genotype × virus interaction: $F_{(1, 36)}$ = 9.299, $P = 0.0043$
Number of platform crossings	Virus and genotype	two-way ANOVA; main effect of genotype: $F_{(1, 36)}$ = 27.52, $P < 0.0001$; main effect of virus: $F_{(1, 36)}$ = 11.81, $P = 0.0015$; genotype × virus interaction: $F_{(1, 36)}$ = 1.604, $P = 0.2135$

Table S5. Statistical analysis of behavioral studies in CRCI female mice infused with Lentivirus in kidney.

Parameter	Comparison	Results
Morris water maze-initial training (*n* = 8)		
Latency to platform	Virus, genotype, and time	three-way ANOVA; main effect of genotype: $F_{(1, 140)} = 82.97$, $P < 0.0001$; main effect of virus: $F_{(2, 140)} = 1.561$, $P = 0.2136$; main effect of time: $F_{(4, 140)} = 131.6$, $P < 0.0001$; time × genotype interaction: $F_{(4, 140)} = 6.876$, $P < 0.0001$; time× virus interaction: $F_{(4, 140)} = 0.4638$, $P = 0.7622$; genotype × virus interaction: $F_{(1, 140)} = 0.3$, $P = 0.5847$; genotype × virus × time interaction: $F_{(4, 140)} = 0.9579$, $P = 0.4328$
Distance traveled to platform	Virus, genotype, and time	three-way ANOVA; main effect of genotype: $F_{(1, 140)} = 56.2$, $P < 0.0001$; main effect of virus: $F_{(1, 140)} = 1.962$, $P = 0.1635$; main effect of time: $F_{(4, 140)} = 123.4$, $P < 0.0001$; time × genotype interaction: $F_{(4, 140)} = 7.394$, $P < 0.0001$; time× virus interaction: $F_{(4, 140)} = 0.7778$, $P = 0.5414$; genotype×virus interaction: $F_{(1, 140)} = 0.637$, $P = 0.4261$; genotype × virus × time interaction: $F_{(4, 140)} = 0.3103$, $P = 0.8707$
Morris water maze-probe trial (*n* =8)		
Percentage time in quadrant	Virus and genotype	two-way ANOVA; main effect of genotype: $F_{(1, 28)} = 74.78$, $P < 0.0001$; main effect of virus: $F_{(1, 28)} = 0.01491$, $P = 0.9037$; genotype × virus interaction: $F_{(1, 28)} = 0.9232$, $P = 0.3449$
Number of platform crossings	Virus and genotype	two-way ANOVA; main effect of genotype: $F_{(1, 28)} = 16.51$, $P = 0.0004$; main effect of virus: $F_{(1, 28)} = 0.3369$, $P = 0.5663$; genotype × virus interaction: $F_{(1, 28)} = 0.9358$, $P = 0.3416$

Table S6. Statistical analysis of behavioral studies in aged female mice after treating with Corn oil/Saline, BBR/Saline, Corn oil/DOX or DOX/BBR.

Parameter	Comparison	Results
Y maze (*n* = 13)	DOX and BBR	two-way ANOVA; main effect of DOX: $F(1, 48) = 19.74$, $P < 0.0001$; main effect of BBR: $F(1, 48) = 5.994$, $P = 0.0181$; DOX × BBR interaction: $F(1, 48) = 4.569$, $P = 0.0377$
Morris water maze–initial training (*n* = 15)		
Latency to platform	DOX, BBR and time	three-way ANOVA; main effect of DOX: $F(1, 240) = 19.58$, $P < 0.0001$; main effect of BBR: $F(1, 240) = 20.68$, $P < 0.0001$; main effect of time: $F(4, 240) = 64.63$, $P < 0.0001$; time × DOX interaction: $F(4, 240) = 3.01$, $P = 0.0189$; time × BBR interaction: $F(4, 240) = 1.356$, $P = 0.2499$; DOX × BBR interaction: $F(1, 240) = 17.05$, $P < 0.0001$; DOX × BBR × time interaction: $F(4, 240) = 1.933$, $P = 0.1056$
Distance traveled to platform	DOX, BBR and time	three-way ANOVA; main effect of DOX: $F(1, 240) = 31.69$, $P < 0.0001$; main effect of BBR: $F(1, 240) = 10.94$, $P = 0.0011$; main effect of time: $F(4, 240) = 92.45$, $P < 0.0001$; time × DOX interaction: $F(4, 240) = 1.519$, $P = 0.1973$; time × BBR interaction: $F(4, 240) = 1.404$, $P = 0.2333$; DOX × BBR interaction: $F(1, 240) = 1.596$, $P = 0.2077$; DOX × BBR × time interaction: $F(4, 240) = 2.942$, $P = 0.0212$
Morris water maze–probe trial (*n* = 15)		
Percentage time in quadrant	DOX and BBR	two-way ANOVA; main effect of DOX: $F(1, 48) = 47.43$, $P < 0.0001$; main effect of BBR: $F(1, 48) = 24.93$, $P < 0.0001$; DOX × BBR interaction: $F(1, 48) = 14.83$, $P = 0.0003$
Number of platform crossings	DOX and BBR	two-way ANOVA; main effect of DOX: $F(1, 48) = 15.54$, $P = 0.0003$; main effect of BBR: $F(1, 48) = 10.67$, $P = 0.0020$; DOX × BBR interaction: $F(1, 48) = 2.854$, $P = 0.0976$;

Supplementary Figures and Figure legends

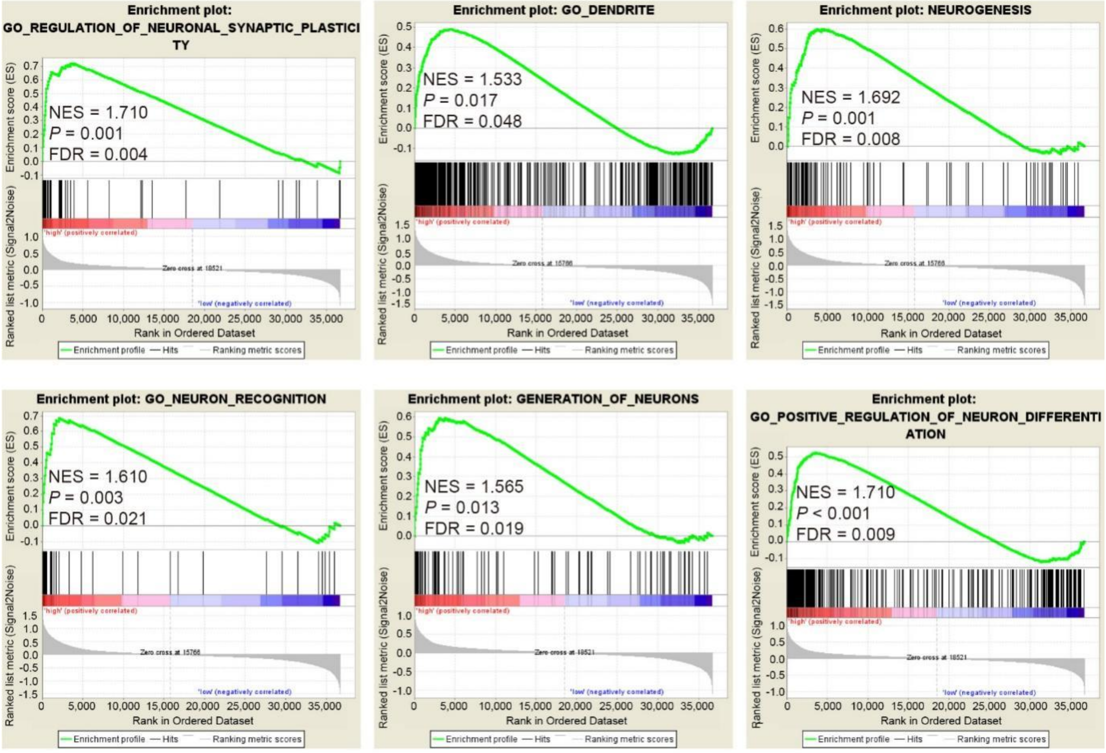

Supplemental Figure 1. PTPRO levels positively correlate with neuron differentiation, plasticity, and neurogenesis. GSEA show positive correlations between PTPRO expression and the neuron differentiation gene signature (GO_POSITIVE_REGULATION_OF_NEURON_DIFFERENTIATION), synaptic plasticity gene signature (GO_REGULATION_OF_NEURONAL_SYNAPTIC_PLASTICITY), neuron recognition gene signature (GO_NEURON_RECOGNITION), neurogenesis gene signature (NEUROGENESIS), neuron generation gene signature (GENERATION_OF_NEURONS) and dendritic formation gene signature (GO_DENDRITE) in a GEO database (GSE29378: hippocampus, n = 31). FDR, false-discovery rate; NES, normalized enrichment score.

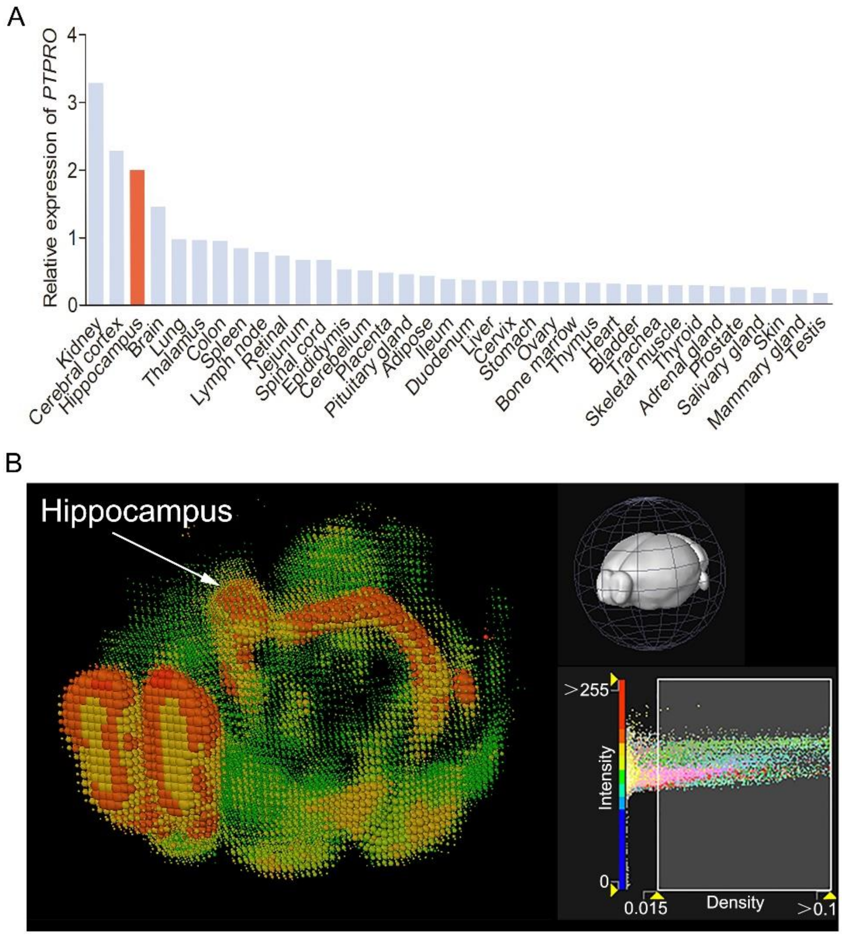

Supplemental Figure 2. Expression of PTPRO in mouse brain is associated with reward processing (Brain Explorer 2). (**A**) PTPRO expression in different human organs (GSE14938). (**B**) Colored spheres represented the expression of PTPRO: Low (blue-green), medium (yellow), and high (red). Spheres also represent each 200 μm^3 voxel, with the size of the spheres directly proportional to the number of objects detected in each voxel. Note that PTPRO is highly expressed in hippocampus. Images and data were obtained from Allen Mouse Brain Atlas (http://mouse.brain-map.org).

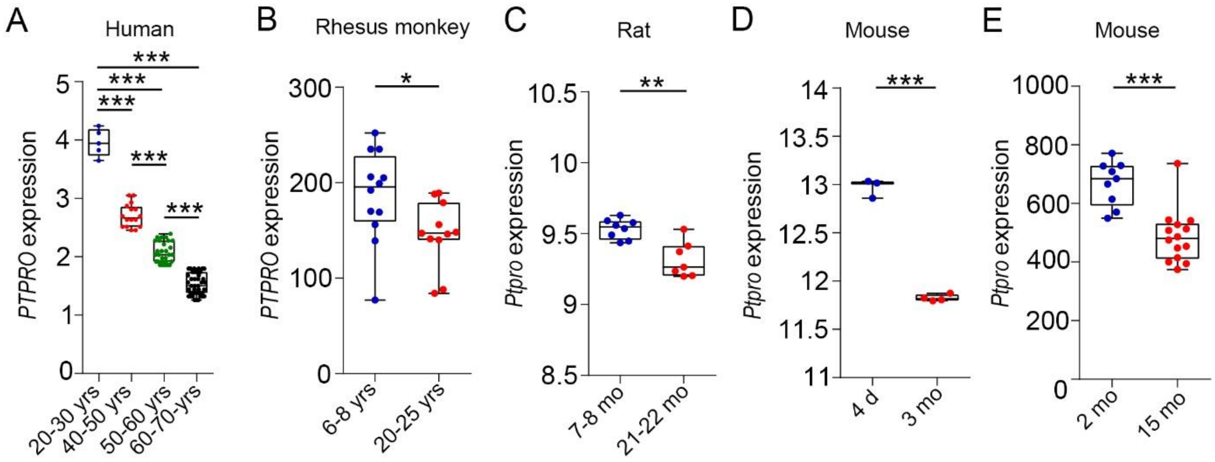

Supplemental Figure 3. Analysis of PTPRO expression in hippocampus using the BrainEXP and GEO data. (**A**) The line chart shows PTPRO expression in hippocampus reduced significantly with age in human. The samples are divided into four groups according to age, with 5 individuals aged 20–30 (mean 24.8) years old, 16 individuals aged 40–50 (mean 45.8) years old, 30 individuals aged 50–60 (mean 56.5) years old and 43 individuals aged 60–70 (mean 65.5) years old. Data are obtained from Brain EXPression Database (BrainEXP) (http://brainexp.org/Search.aspx). (**B**) Data from GEO database illustrate that the expression of PTPRO in hippocampi of young rhesus is higher than the old (GSE11697: young, n = 12, 6-8 years old; old, n = 11, 20-25 years old). (**C**) Data from GEO database illustrate that the expression of PTPRO in hippocampi of young rats is higher than the old (GSE20219: young, n = 8, 7-8 months old; old, n = 7, 21-22 months old). (**D**) Data from GEO database illustrate that the expression of PTPRO in hippocampi of newborn mice is higher than the adult (GSE21718: newborn, n = 3, 4 days old; adult, n = 4, 3 months old). (**E**) Data from GEO database illustrate that the expression of PTPRO in hippocampi of young mice is higher than the middle-aged (GSE5078: young, n = 9, 2 months old; middle-aged, n = 14, 15 months old). $^{*}P<0.05$, $^{**}P<0.01$, $^{***}P<0.001$ by student's t test (**B**-**E**) or one-way ANOVA followed by a Tukey–Kramer *post-hoc* test (**A**).

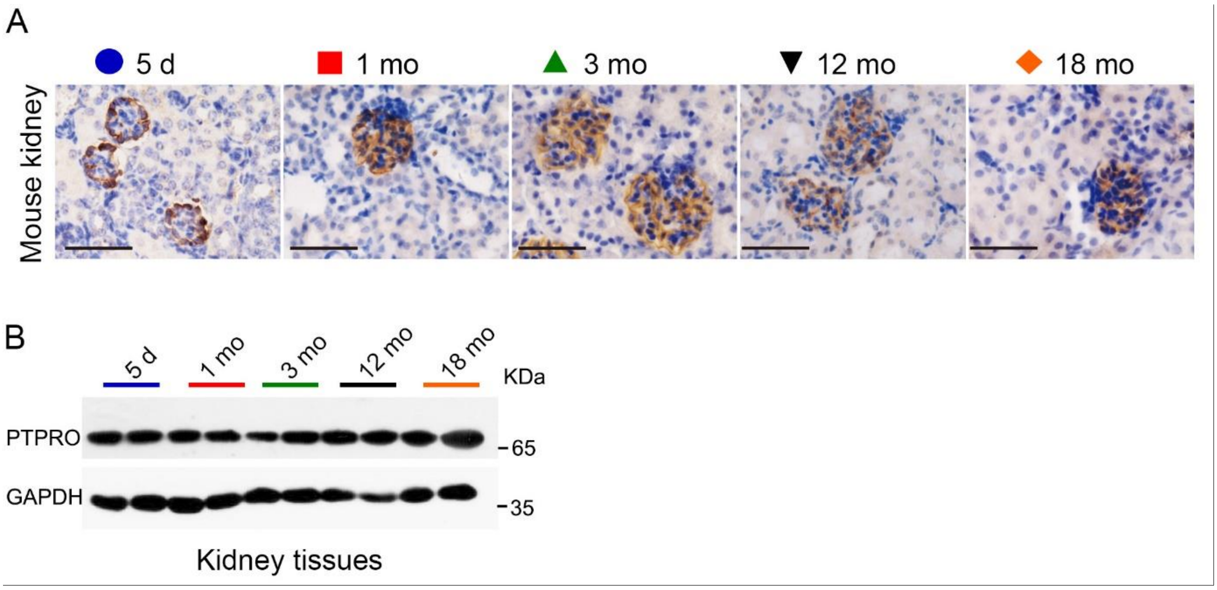

Supplemental Figure 4. The expression of PTPRO in the mice kidney at the indicated ages. (**A**) Representative IHC images of PTPRO in mouse kidney, which was no detectable age-dependent change. Scale bars: 50 µm. (**B**) The protein levers of mouse kidney PTPRO estimated by immunoblotting. *n* = 6 mice per age group with equal sex ratio. The data represent three independent experiments.

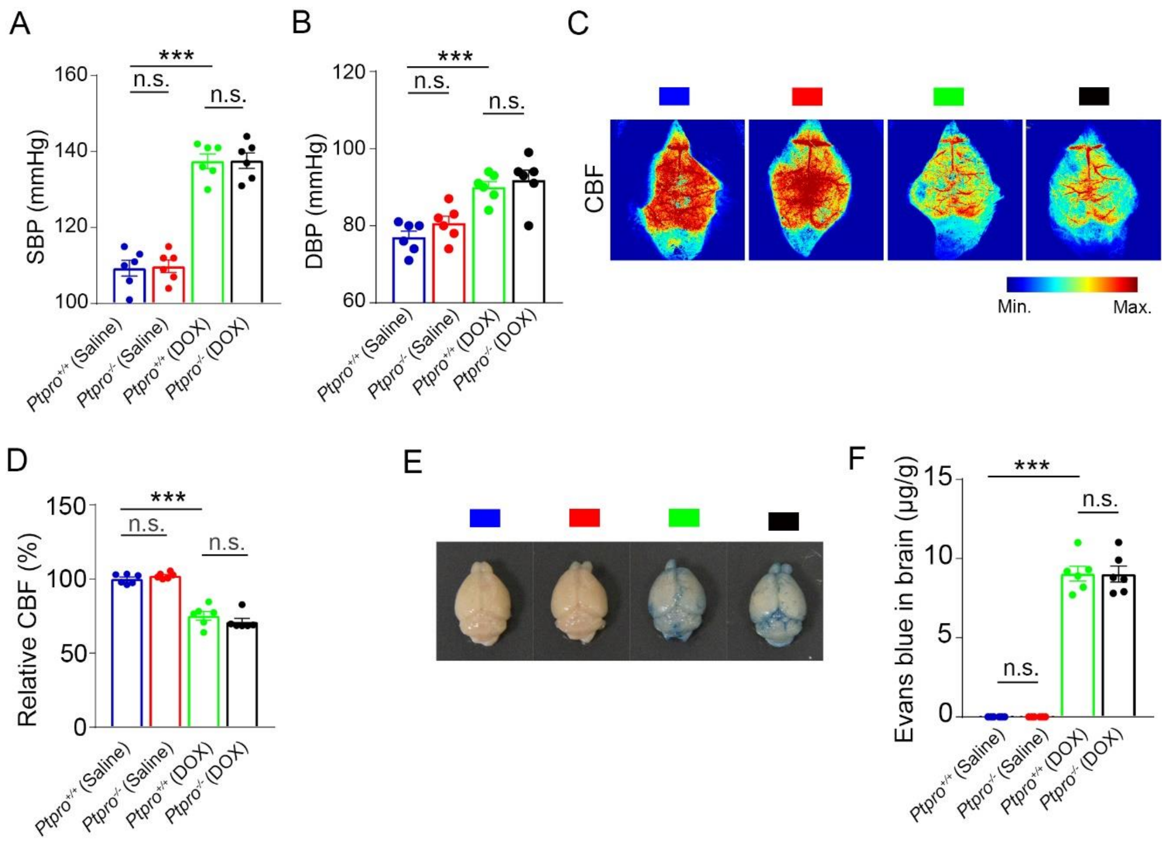

Supplemental Figure 5. *Ptpro* deletion does not affect the blood pressure, CBF and BBB integrity in the CRCI mouse model. (**A** and **B**) Systolic blood pressure (SBP) (**A**) and diastolic blood pressure (DBP) (**B**) were measured by the tail-cuff method. (**C**) Representative images of CBF perfusion in four groups of mice. (**D**) Quantitative analysis of the level of CBF perfusion in four groups of mice. (**E**) Representative dorsal view of mouse brains injected with Evans blue dye. (**F**) Quantitative analysis of Evans blue extravasation in brain tissue using spectrophotometry at 620 nm. n = 6 per group. The data represent three independent experiments. Error bars: SEM. n.s., not significant; $^{***}P<0.001$ by one-way ANOVA followed by a Tukey–Kramer *post- hoc* test.

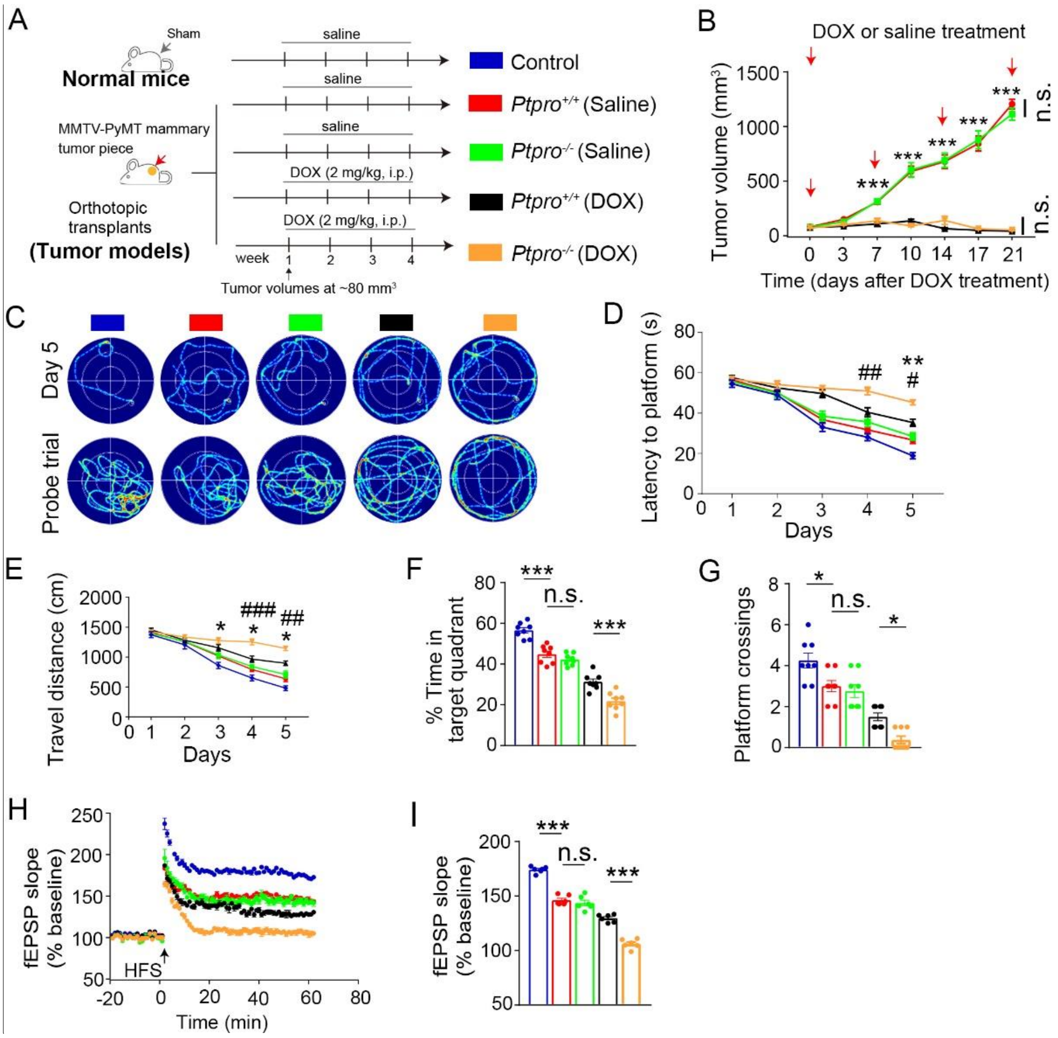

Supplemental Figure 6. *Ptpro* deletion increases DOX-induced CRCI in the MMTV-PyMT breast cancer model. (**A**) Schematic of the experimental design. (**B**) DOX treatment reduces MMTV-PyMT tumor volume in FVB female mice. *Ptpro* deletion barely altered MMTV-PyMT tumor growth in FVB female mice. Mice were given DOX or saline when tumor volume reached approximately 80 mm^3 (indicated by red arrows). (**C**) Representative swimming traces in MWM test. (**D** and **E**) Training trials were performed in the MWM test. The time taken to reach the submerged platform (**D**) and the distances traveled before reaching the submerged platform (**E**), n = 8 per group. (**F** and **G**) A probe trial was performed in the MWM test. Time spent in the target quadrant (**F**) and the number of crossings before reaching the target location (**G**), n = 8 per group. (**H**) Time course of fEPSP measures were recorded in the hippocampal CA1 region before and after 100-Hz stimulation in the Schaffer collateral region. Normalized fEPSP slopes were plotted every 1 minute for each group. (**I**) The averaged fEPSPs recorded 56–60 minutes after induction of LTP. n = 6 slices from 3 mice in each group. The data represent three independent experiments. Error bars: SEM. n.s., not significant; *Saline-treated *Ptpro*$^{+/+}$ mice *vs.* DOX-treated *Ptpro*$^{+/+}$ mice, $^*P$<0.05, $^{**}P$<0.01, $^{***}P$<0.001 by student's t test ; *Control *vs.* Saline-treated *Ptpro*$^{+/+}$ mice, $^*P$<0.05, $^{**}P$<0.01, $^{***}P$<0.001 by student's t test (**D**-**I**); $^\#$DOX-treated *Ptpro*$^{+/+}$ mice *vs.* DOX-treated *Ptpro*$^{-/-}$ mice, $^\#P$<0.05, $^{\#\#}P$<0.01, $^{\#\#\#}P$<0.001, $^*P$<0.05, $^{***}P$<0.001 by two way ANOVA (**F**, **G** and **I**)and three-way ANOVA (**B**, **D** and **E**) followed by a Tukey-Kramer *post-hoc* test. All values and statistical analysis of behavioral experiments are provided in Supplementary Table S2.

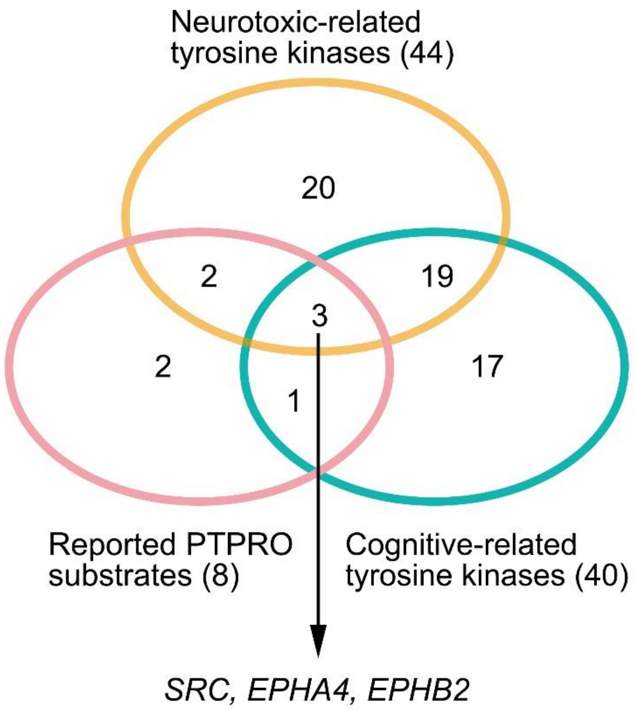

Supplemental Figure 7. The Venn diagram shows the overlap of reported PTPRO substrates, cognitive-related tyrosine kinases, and neurotoxic-related tyrosine kinases. Genes classified as cognitive-relevant tyrosine kinases and neurotoxic-related tyrosine kinases were annotated in GSEA website (http://software.broadinstitute.org/gsea/index.jsp). The overlapping genes of these three sets are *SRC*, *EPHA4* and *EPHB2*.

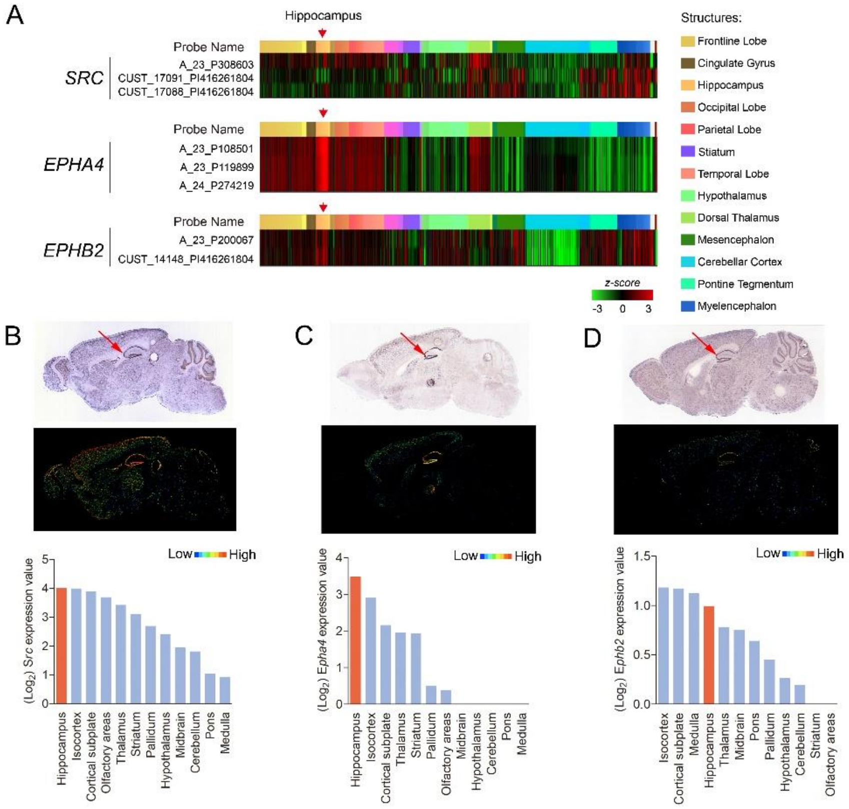

Supplemental Figure 8. PTPRO substrates (SRC, EPHA4, and EPHB2) are grouped by brain regions. (**A**) Gene expression in human is shown as individually normalized gene expression (Z-scores normalized to whole human brain expression). The heat maps show scores across the whole human brain and for each of the six subjects contained in the database beside each other, where red indicates high expression and green indicates low expression. Red arrows indicate hippocampus. Images and data are obtained from BrainSpan (http://www.brainspan.org/lcm/search?search_type=user_selections). (**B-D**) *Src* (**B**), *Epha4* (**C**) and *Ephb2* (**D**) expression were detected in the mouse brain. Upper panel show representative ISH staining images in mouse brain (Sagittal). The middle panel shows the expression levels in the mouse brain, red indicates high expression and blue indicates low expression. Bottom panel shows the quantification of region-specific expression of three genes in the mouse brain. Red indicates hippocampus. Images and data were obtained from Allen Mouse Brain Atlas (http://mouse.brain-map.org).

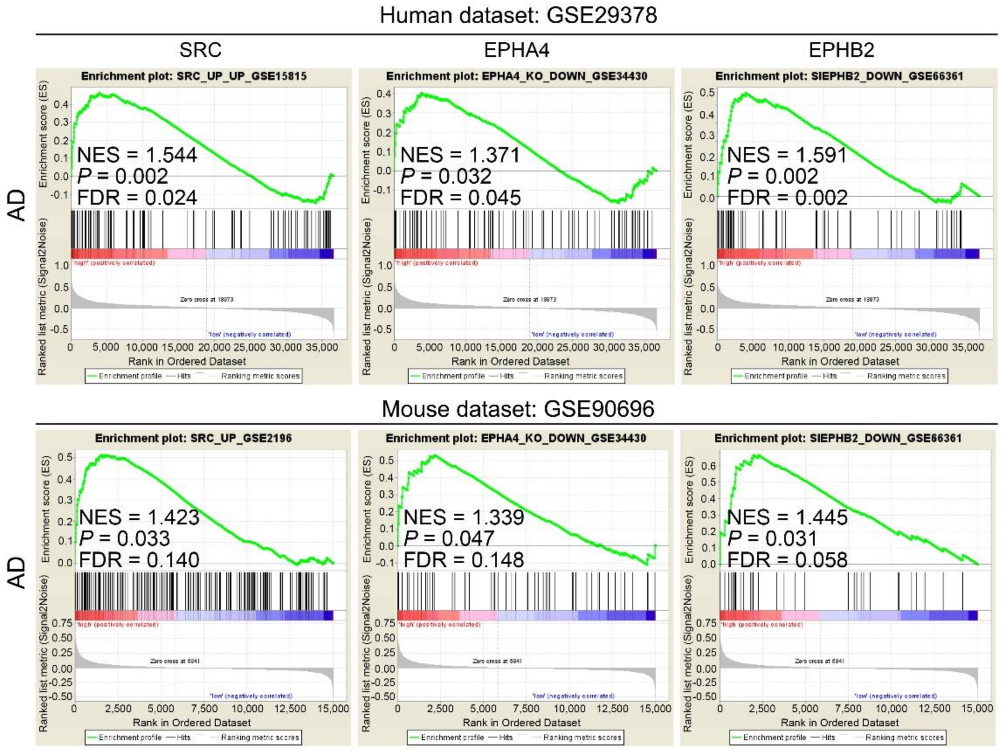

Supplemental Figure 9. Correlations between PTPRO substrates (SRC, EPHA4, and EPHB2) and cognitive impairment. GSEA reveals that SRC, EPHA4, and EPHB2 signatures positively correlate with cognitive impairment in a published human dataset (GSE29378: normal hippocampus, *n* = 32; AD hippocampus, *n* = 31, upper panel) and a published mouse dataset (GSE90696: normal hippocampus, n = 20; AD hippocampus, *n* = 18, bottom panel). FDR, false-discovery rate; NES, normalized enrichment score.

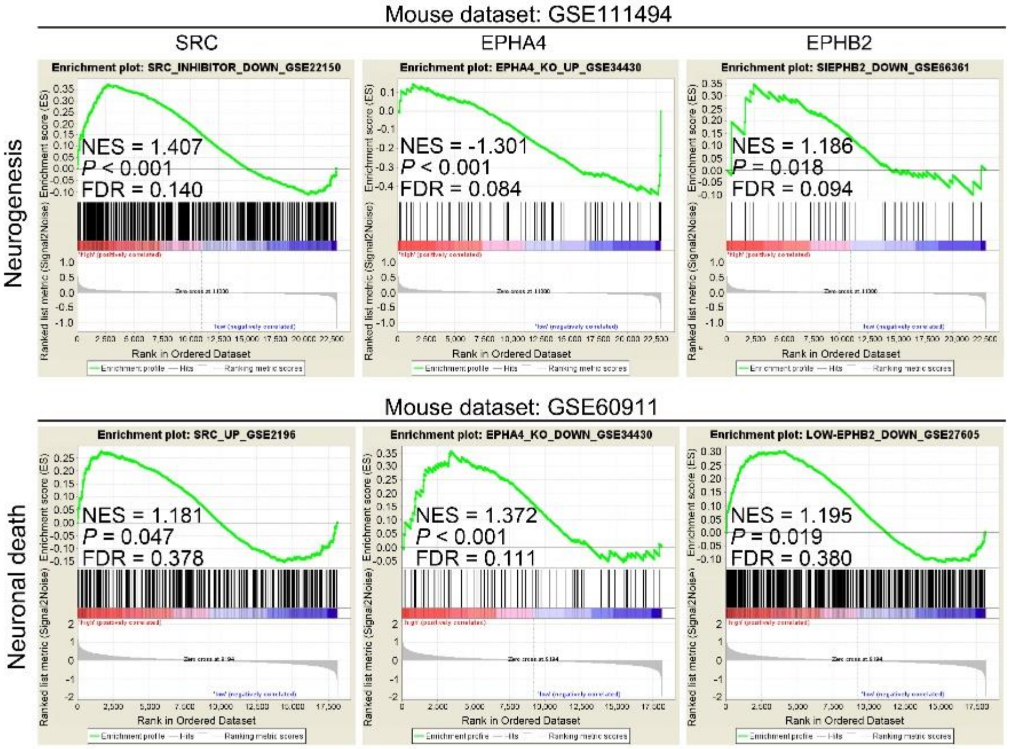

Supplemental Figure 10. Correlations between PTPRO substrates (SRC, EPHA4, and EPHB2) and neurogenesis and neuronal death. GSEA reveals that SRC, EPHA4, and EPHB2 negatively correlated with neurogenesis in a published mouse dataset (GSE111494: normal neurogenesis, *n* = 3; neurogenesis inhibition, *n* = 3, upper panel) and positively correlated with neuronal death in a published mouse database (GSE60911: normal, *n* = 4; neuronal death, *n* = 4, bottom panel). FDR, false-discovery rate; NES, normalized enrichment score.

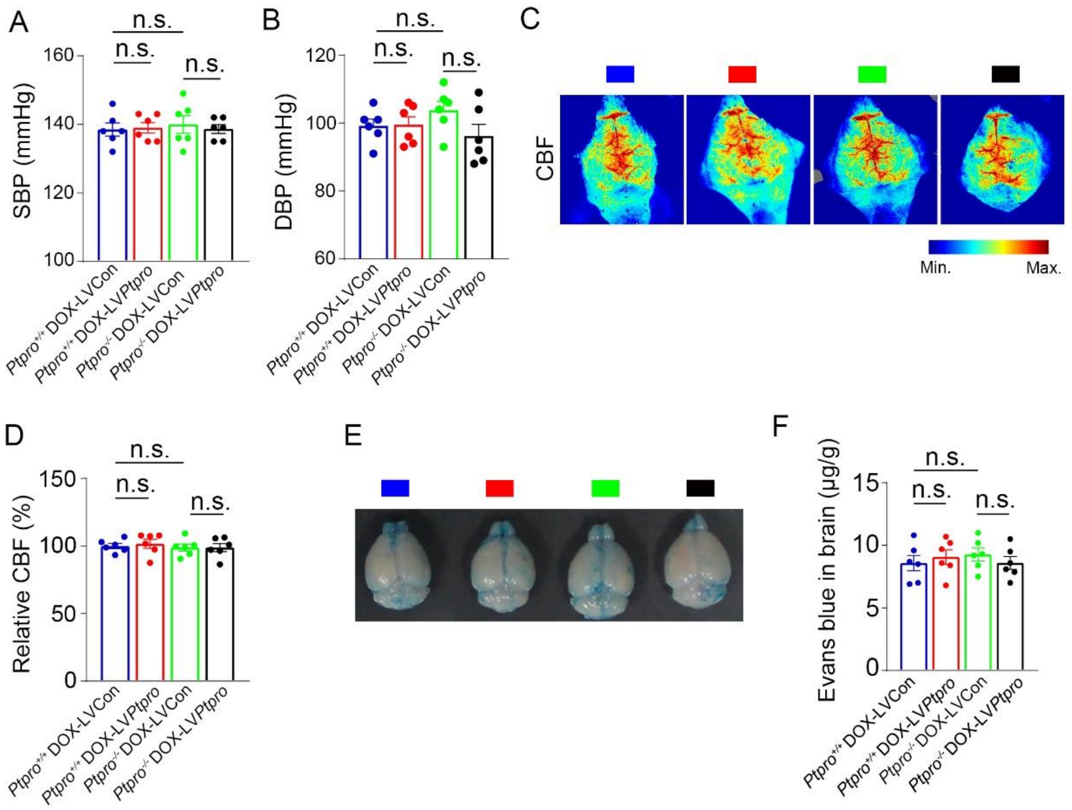

Supplemental Figure 11. Region-specific restoration of hippocampal PTPRO does not affect the blood pressure, CBF and BBB integrity in the CRCI mouse model. (**A** and **B**) SBP (**A**) and DBP (**B**) were measured by the tail-cuff method. (**C**) Representative images of CBF perfusion in four groups of mice. (**D**) Quantitative analysis of the level of CBF perfusion in four groups of mice. (**E**) Representative dorsal view of mouse brains injected with Evans blue dye. (**F**) Quantitative analysis of Evans blue extravasation in brain tissue using spectrophotometry at 620 nm. *n* = 6 per group. The data represent three independent experiments. Error bars: SEM. n.s., not significant; One-way ANOVA followed by a Tukey-Kramer post-hoc test was used to analyze the data.

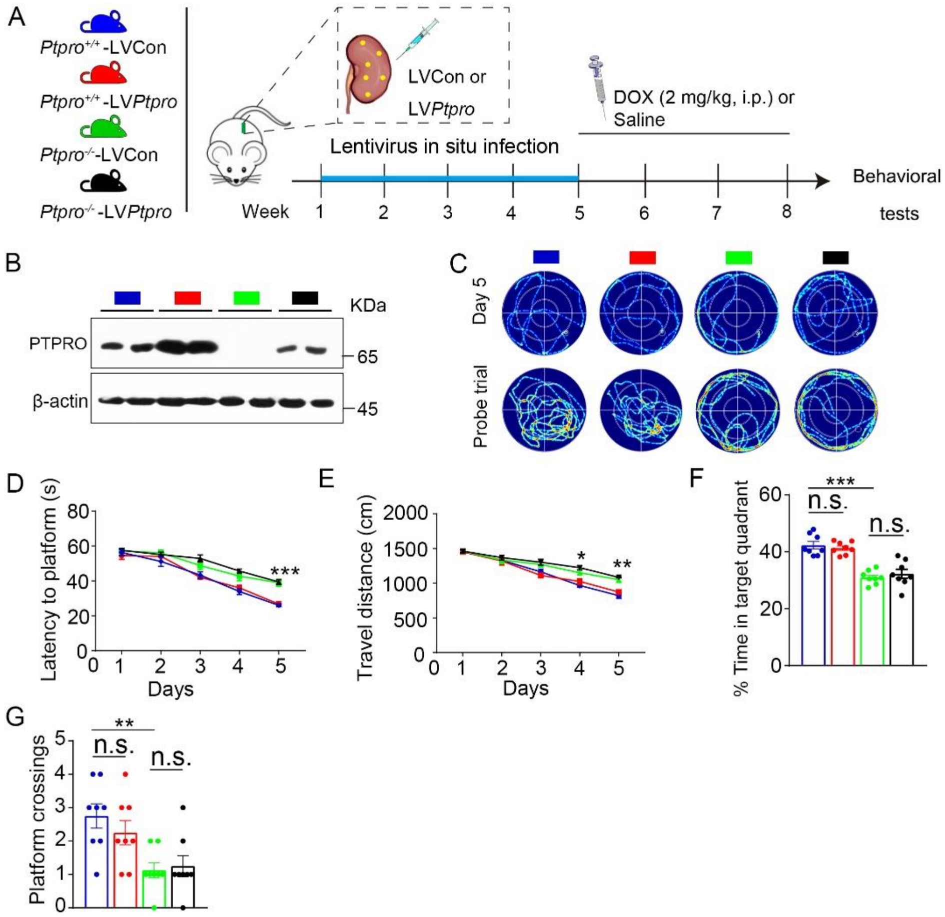

Supplemental Figure 12. Region-specific restoration of kidney PTPRO does not affect cognitive function. (**A**) Schematic of the experimental design. (**B**) Immunoblotting of PTPRO in indicated groups. (**C**) Representative swimming traces in MWM test. (**D**-**E**) Training trials were performed in the MWM test. The time taken to reach the submerged platform (**D**) and the distances traveled before reaching the submerged platform (**E**), $n = 8$ per group. (**F**-**G**) A probe trial was performed in the MWM test. Time spent in the target quadrant (**F)** and the number of crossings before reaching the target location (**G**), $n = 8$ per group. The data represent three independent experiments. Error bars: SEM. n.s., not significant; **Ptpro*$^{+/+}$-LVCon *vs*. *Ptpro*$^{-/-}$-LVCon, $^*P$<0.05, $^{**}P$<0.01, $^{***}P$<0.001 by two-way ANOVA (**F** and **G**) and three-way ANOVA (**D** and **E**) followed by a Tukey-Kramer *post-hoc* test. All values and statistical analysis of behavioral experiments are provided in Supplementary Table S5.

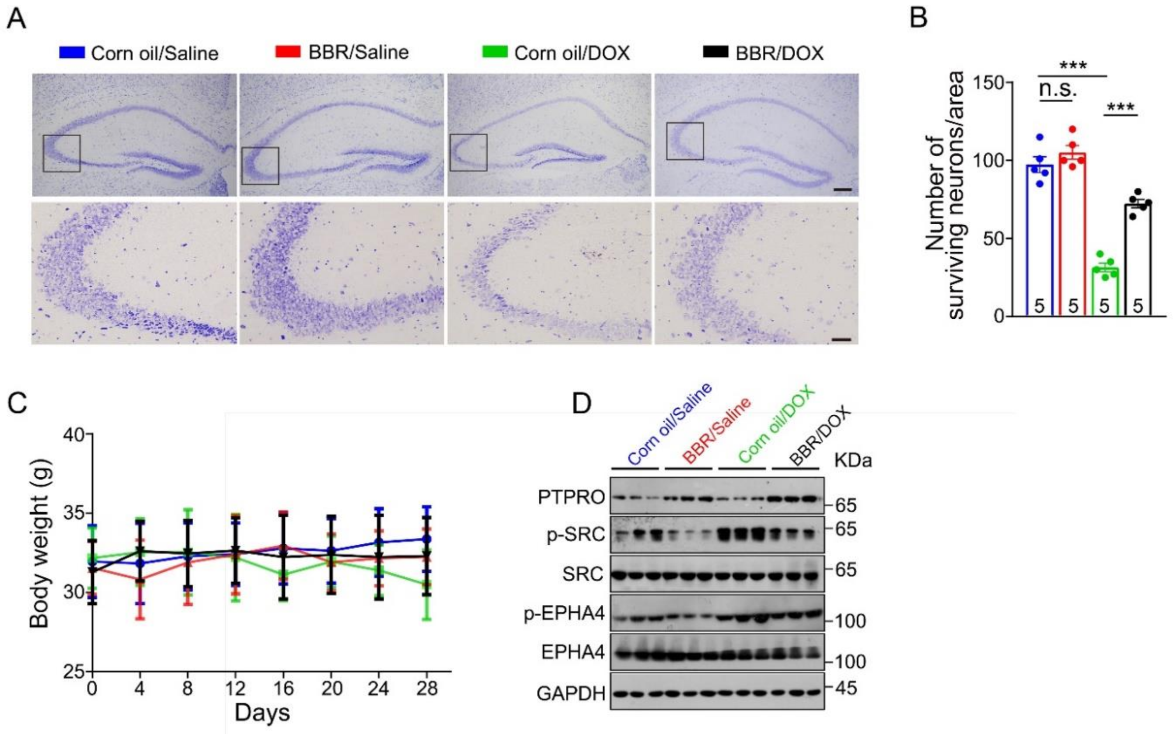

Supplemental Figure 13. Protective effects of BBR on DOX-induced hippocampal damage and absence of side effects in aged female mice with BBR treatment. (**A**) Nissl staining of the hippocampus (upper panel) and hippocampal CA3 region (bottom panel) of mice subjected to different treatments. Scale bars: 200 µm (upper panel), 50 µm (bottom panel). The data represent three independent experiments. (**B**) Quantification of surviving neurons in the hippocampal CA3 region of mice subjected to different treatments. $n = 5$ mice per group. (**C**) During the BBR treatment, no significant changes were found in the bodyweight of the mice ($n = 13$ per group). (**D**) Immunoblotting of PTPRO, p-SRC, SRC, p-EPHA4 and EPHA4 in the hippocampi of mice with different treatments. Error bars: SEM. n.s., not significant; $^{***}P < 0.001$ by two-way ANOVA followed by a Tukey-Kramer *post-hoc* test.

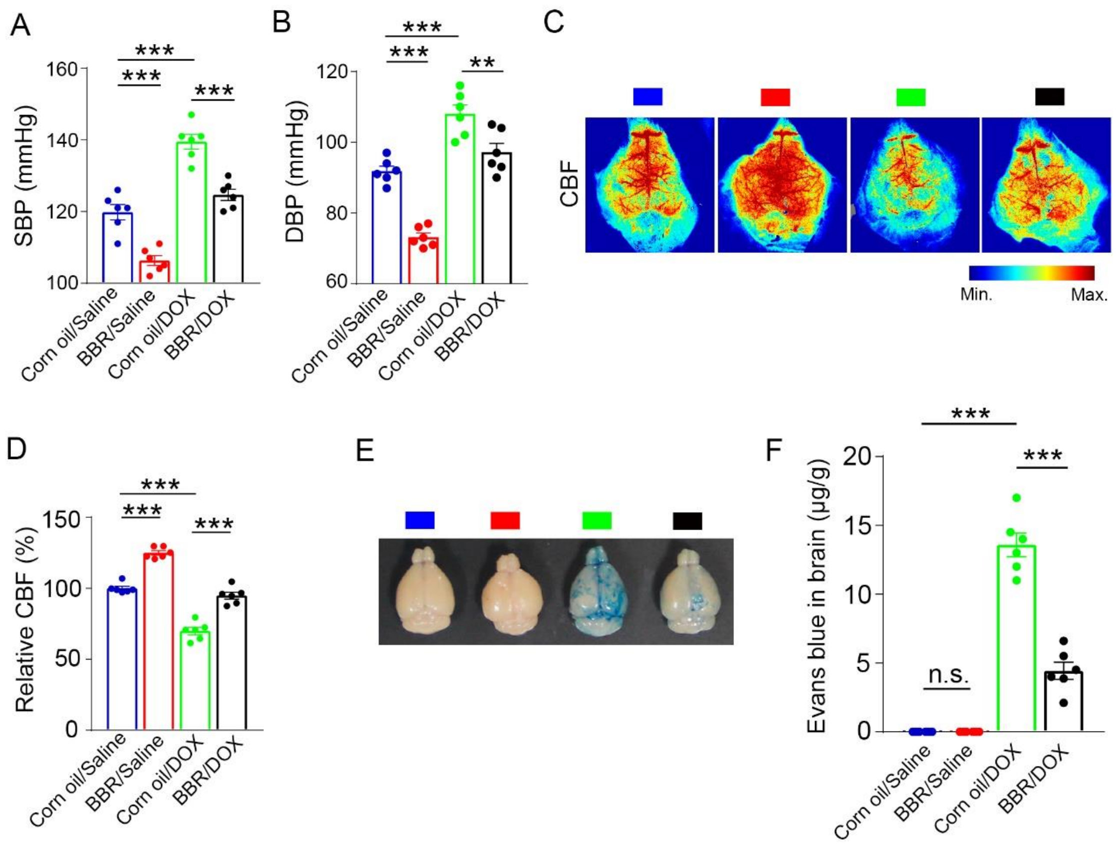

Supplemental Figure 14. BBR plays a protective role against DOX-induced BBB damage, systolic and diastolic blood pressure elevation, and CBF reduction. (**A** and **B**) SBP (**A**) and DBP (**B**) were measured by the tail-cuff method. (**C)** Representative images of CBF perfusion in four groups of mice. (**D**) Quantitative analysis of the level of CBF perfusion in four groups of mice. (**E**) Representative dorsal view of mouse brains injected with Evans blue dye. (**F**) Quantitative analysis of Evans blue extravasation in brain tissue using spectrophotometry at 620 nm. *n* = 6 per group. The data represent three independent experiments. Error bars: SEM. $^{**}P<0.01$, $^{***}P<0.001$ by one-way ANOVA followed by a Tukey-Kramer *post-hoc* test.

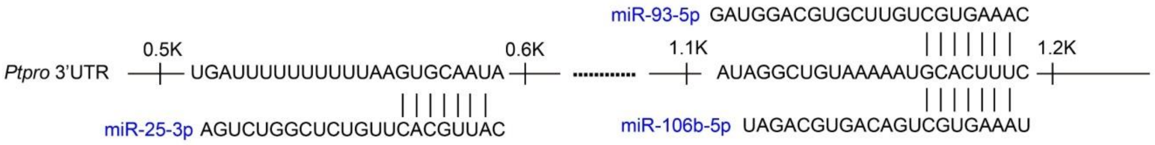

Supplemental Figure 15. The potential binding sites of miR-25-3p, miR-93-5p and miR-106b-5p in the 3'-UTR of *Ptpro* mRNA. The potential binding sites of miR-25-3p, miR-93-5p and miR-106b-5p in 3′ UTR of *Ptpro* is predicted by Targetscan.

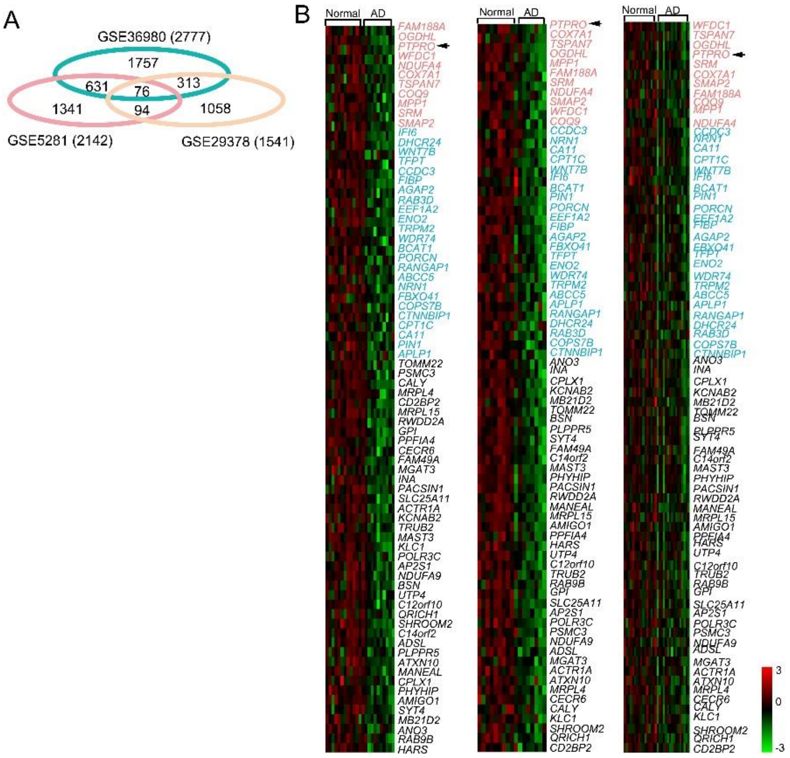

Supplemental Figure 16. PTPRO is downregulated in the hippocampi of patients with cognitive disorders. (**A**) The Venn diagram shows downregulated genes in the hippocampi of AD from three GEO datasets (GSE5281: control, n = 13, AD, n = 10; GSE36980: control, n = 10, AD, n = 7; GSE29378: control, n = 16, AD, n = 16). Numbers in the overlapping region of the Venn diagram represents commonly downregulated genes. (**B**) The heat maps show the expression of the 76 genes in AD patients and control. The tumor suppressors were shown in red, oncogenes in blue, and the others in black.

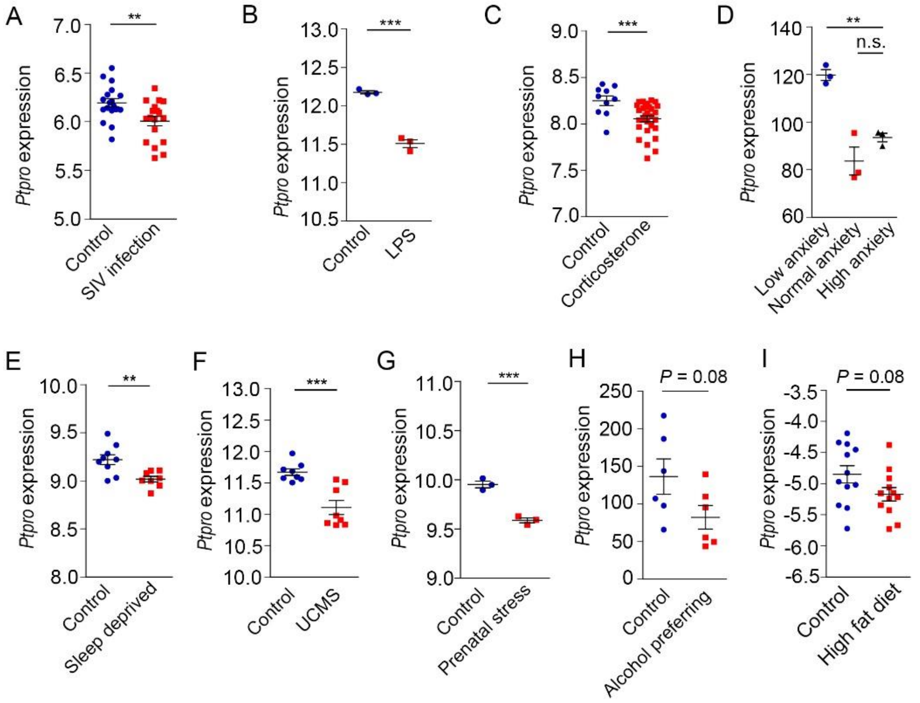

Supplemental Figure 17. Relationship between hippocampal PTPRO expression and risk factors in conditions both cancer susceptibility and cognitive deficiency. (**A**) PTPRO expression decreases in rhesus hippocampus of simian immunodeficiency virus (SIV) infected group (n = 18) compared with that of control group (n = 18) (GSE13824). (**B**) PTPRO expression decreases in epileptic mouse hippocampus after 4-hour LPS treatment (systemic inflammation, n = 3) compared with control group (n = 3) (GSE81024). (**C**) PTPRO expression decreases in rat hippocampus of corticosterone treatment group (n = 29) compared with that of control group (n = 10) (GSE37618). (**D**) PTPRO expression decreases in mouse hippocampus of high anxiety group (n = 3) and normal anxiety group (n = 3) compared with that of low anxiety group (n = 3) (GSE29015). (**E**) PTPRO expression decreases in mouse hippocampus of sleep deprivation group (n = 8) compared with control group (n = 9) (GSE33302). (**F**) PTPRO expression decreases in mouse hippocampus exposed unpredictable chronic mild stress (UCMS) (n = 8) compared with control group (n = 8) (GSE84183). (**G**) PTPRO expression decreases in mouse hippocampus exposed prenatal stress (n = 3) compared with control group (n = 3) (GSE26025). (**H**) PTPRO expression decreases in rat hippocampus of alcohol-preferring group (n = 6) compared with that of non-alcohol preferring group (n = 10) (GSE4494). (**I**) PTPRO expression decreases in mouse hippocampus exposed high-fat diet (n = 12) compared with that of low-fat control diet (n = 12) (GSE63174). Error bars: SEM. n.s., not significant; $^{**}P<0.01$, $^{***}P<0.001$ by Student's t test (**A-C** and **E-I**) and $^{**}P<0.01$ by one-way ANOVA followed by a Tukey–Kramer *post- hoc* test (**D**).
